# Quantifying cross-sectional and longitudinal associations in mental health symptoms within families: network models applied to UK cohort data

**DOI:** 10.1136/bmjopen-2025-104829

**Published:** 2025-10-06

**Authors:** Yushi Bai, Archie Rayner, Kathryn M. Abel, Sam Cartwright-Hatton, Ming Wai Wan, Matthias Pierce

**Affiliations:** 1 Centre for Women’s Mental Health, Faculty of Biology, Medicine and Health Sciences, University of Manchester, Manchester, UK; 2 Imperial College London, London, UK; 3 Greater Manchester Mental Health NHS Foundation Trust, Manchester, UK; 4 School of Psychology, University of Sussex, Brighton, UK; 5 Division of Psychology and Mental Health, School of Health Sciences, Faculty of Biology, Medicine and Health Sciences, The University of Manchester, Manchester, UK

**Keywords:** Public health, MENTAL HEALTH, Family, Child & adolescent psychiatry

## Abstract

**Abstract:**

**Objectives:**

Families offer promising targets for mental health interventions. Existing evidence investigates parent-child dyads or partners; we use an innovative approach to look at triads of parents and their children. This gives us more detail on mental health dimensions and individuals central to mental health transmission in families.

**Design:**

Both cross-sectional and longitudinal network models

**Setting:**

We identified triads of children (under age 16), mothers and fathers from the UK Household Longitudinal Study, between 2009 and 2022.

**Participants and methods:**

Cross-sectional networks captured independent associations between family members’ mental health (n=8795 families). Longitudinal networks examined directional temporal associations among family members’ emotional symptoms (n=3757 families).

**Primary outcome measures:**

Children’s and parents’ mental health dimensions were assessed using the Strengths and Difficulties Questionnaire and the General Health Questionnaire, respectively.

**Results:**

Mothers’ mental health, particularly emotional symptoms, was linked to children’s mental health, while fathers’ symptoms showed no independent association. In the longitudinal network, maternal feelings of being overwhelmed were associated with children’s future worry, affecting symptoms of nervousness and unhappiness, which then fed back into worsening maternal emotional symptoms.

**Conclusions:**

Investigating family mental health using network models highlights mothers’ central role. The longitudinal relationship between maternal feelings of being overwhelmed and children’s anxiety, and the subsequent feedback into maternal anxiety, indicates a promising target for intervention.

**STRENGTHS AND LIMITATIONS OF THIS STUDY**

- We used network models that enabled us to consider multidimensional family mental health processes through the simultaneous inclusion of both multiple mental health domains and family members.
- Longitudinal network analyses allowed us to map the directional pathways of emotional symptoms over time, allowing for more robust conclusions.
- Although multiple mental health dimensions were considered, externalising symptoms were not available for parents.
- Our analysis was restricted to triads where mothers and fathers could be identified and is therefore not generalisable to single parent or same-sex parent families.

## Background

 Family is a dynamic, interconnected and multifaceted system in which members are interdependent and unavoidably embedded. This family system theory implies the interdependence of members in the family system, meaning that the mental health and well-being of one member can have a profound effect on another (for better or worse).^1^ Children’s mental health, in particular, is formed by, and within, their family: through shared genes,^2^ nurturing behaviours of caregivers^3^ and sibling dynamics.^4^ These processes interact with each other and with the wider housing, economic and social ecosystem within which families live.^5^

Given the family’s central role in shaping and sustaining mental health, interventions and policies should consider how the family mental health ecosystem operates. In particular, there may be targets within the family that could improve children and young people’s mental health—a public health priority because of its well-publicised decline in recent decades.^6^ Emotional disorders in young people, for example, have shown not only increasing prevalence among recent cohorts, but also earlier onset,^6^ underscoring the need for early intervention and prevention strategies. As the immediate environment that shapes and maintains young people’s mental health, it is reasonable to assume the family serves as a key instrument of change for this. This is supported by the effectiveness of family-based approaches (eg, family therapy, parent training) for families of young people with common mental health problems (eg, mood disorders, externalising behaviours) where children are the primary focus of concern.^7^ However, to optimise interventions, whether at the public health level or for individuals, a better understanding is needed of which family relationships and dimensions of mental health are most important for effecting positive change in the mental health of both children and their parents. Additionally, tailoring these towards families requires a detailed understanding of how external factors, such as socioeconomic status, moderate any within-family relationships.

There are several challenges in understanding how mental health functions within families. First, family mental health is multidimensional: mental health domains exist within individuals and individuals exist within families. Most studies focus on isolated relationships, usually mother-child pairs, potentially missing the role that partners or siblings may play in family mental health.^8^ Second, even when family processes are considered, previous research often conceptualises this as family functioning (eg, family flexibility, cohesion and communication), or as the family’s emotional climate influencing the child’s mental health.^9^ While useful in showing the impact of the family ecosystem, it does not necessarily identify what components are central to the transmission of risk across family members.

We address these challenges using a ‘network approach’ to model mental health dynamics between family members. Network models characterise mental health symptoms as ‘nodes’, and the edges connecting these nodes reflect independent associations between the nodes.^10^ It is based on the idea that a higher-level concept (family mental health in our case) arises from lower-level processes (symptoms in individual family members).^10^ This makes it a powerful tool for estimating and graphically representing dynamic, multivariate systems with clear visualisation of connected elements within those systems, and quantification of the inter-relationships between variables. A network approach has previously been used to explore the care systems that modern adolescents navigate^11^ and to examine cross-sectional associations between depression symptoms in parent pairs and their association with later emotional problems in their offspring.^12^ However, since the study relied on cross-sectional data, it was limited in its capacity to assess feedback mechanisms between family members.

By using mental health measures from children and parents, we first aim to describe unique associations between broad domains of family members’ mental health using cross-sectional networks. This was intended to provide an overall picture of the family mental health network structure. Young people have experienced a sharp increase in emotional symptoms, with a relative rise of 63% from 2004 to 2017,^13^ while trends in other symptoms (eg, conduct behaviours) have been less pronounced or remained relatively stable.^14^ Therefore, we then focus on specific symptoms of emotional problems using a longitudinal network to model a directional network between family members. This allowed us to model the dynamic pathways through which emotional symptoms may emerge and evolve in the family context. To our knowledge, the longitudinal network model has not been used before to examine family mental health. To identify potential intervention targets, we aim to determine key dimensions of mental health and key individuals who play dominant roles in the transmission of risk between family members. Furthermore, we explore differences in family mental health symptom networks from different subgroups, according to children’s sex, age and family socioeconomic status.

## Methods

### Data

This study used data from the UK Household Longitudinal Study (UKHLS, also known as ‘Understanding Society’, data DOI: 10.5255/UKDA-SN-6614-20), an ongoing, nationally representative longitudinal survey of UK households.^15^ The survey collects annual data (‘waves’) from each adult household member, including demographics, mental health and socioeconomic characteristics. This study uses data from waves 1–13 spanning 2009–2010 to 2021–2022.

Given our aim to explore transmission pathways between both parents and children, ‘families’ were identified using triads of children (under age 16) and two parents, defined as an adult woman and a man living in the same household (whether birth parents or not). This resulted in an overall 8795 families included in the analysis, accounting for around 75.6% of households with children. Parents’ self-reported mental health was collected across waves 1–13, while parent-reported mental health for children under 10 was based on waves 3 to 13 (2011–2022). Self-reported mental health data for adolescents (10–15 years) were available only in waves 1, 3, 5, 7, 9, 11 and 13. Children’s sex, age and monthly household income were recorded in each wave, enabling subgroup network analysis.

### Mental health measures

The Strengths and Difficulties Questionnaire (SDQ)^16^ was used to measure children (under 10 years, parent-report) and young people’s (10–15 years, self-report) mental health symptoms. The SDQ is a 25-item instrument with a 3-point response scale, measuring five domains: emotional symptoms, peer relationships, conduct problems, hyperactivity or inattention and pro-social behaviours. Each domain was assessed using five items. Parents’ mental health symptoms were measured using the General Health Questionnaire-12 (GHQ-12),^17^ a 12-item instrument with a 4-point response scale.

For the cross-sectional model, associations between broad dimensions of mental health within families were examined, requiring dimension reduction to avoid a model with too many measures. Thus, the SDQ was categorised into three dimensions^18^: internalising (emotional and peer problems, 10-items); externalising (conduct problems and hyperactivity, 10-items) and pro-social behaviours (5-items); and the GHQ-12 measures general mental distress and, according to Shevlin and Adamson,^19^ can be divided into three subscales to reflect: emotional symptoms (4-items), social dysfunction (6-items) and loss of confidence (2-items). Summed scores were used, with higher scores indicating poorer mental health.

For the longitudinal network analysis, the focus was specifically on how emotional problems were transmitted within families; therefore, we conducted the analysis at the item level. For young people, the five SDQ items representing emotional problems were assessed: unhappy (‘often unhappy, down-hearted or tearful’), worry (‘worry a lot’), nervous (‘nervous in new situations’), fears (‘have many fears, easily scared’) and somatic (‘a lot of headaches, stomach-aches or sickness’); and for adults the four GHQ-12 items representing emotional symptoms were examined: unhappy (‘unhappy or depressed’), worry (‘lost sleep over worry’), strain (‘constantly under strain’) and overwhelmed (‘could not overcome difficulties’). Higher scores on each item indicate more severe emotional symptoms.

### Statistical analysis: cross-sectional network on broad family mental health dimensions

Cross-sectional network models were fitted, with three mental health dimensions in children and three in both parents represented as nodes. Edges between nodes reflected partial correlations, indicating conditionally independent associations after controlling for all other nodes in the network. Data from all available waves were used, with single observations randomly selected for families with repeated measures. This approach allowed us to maximise sample size while maintaining temporal independence. We included all children and young people who met the selection criteria, including multiple children from the same family, by randomly selecting one observation for each child. 79.8% of children in the sample came from different families. We conducted a sensitivity analysis by randomly selecting only one child per family. The results remained largely consistent with the main findings (see online supplemental appendix S1), supporting the validity of the current approach.

Derived variables based on the sum scores of the SDQ and GHQ-12 items were treated as continuous, in line with empirical evidence that aggregated Likert scales approximate continuous distributions.^20 21^ The marginal distributions of all variables were visualised and are provided in online supplementary appendix figure S2. Some approximated normality (eg, children’s externalising behaviours), whereas others deviated from it (eg, parents’ loss of confidence). Given that parametric tests remain sufficiently robust when analysing Likert-scale data despite violations of the normality assumption,^21 22^ we retained a Gaussian specification for all variables. Networks were fitted using Mixed Graphical Models using the ‘mgm’ package in R. Spurious associations were controlled using the least absolute shrinkage and selection operator (Lasso) penalty, which shrinks edges with small weights to zero. All other parameters are set to their default values. Only edges that had non-zero values for both directions (eg, A regressed on B and B regressed on A) were included. Networks were visualised using the ‘qgraph’ R-package, with shorter, thicker edges for highly connected nodes and blue/red edges for positive/negative associations.

The stability of the network was tested using the correlation stability (CS) coefficient, estimated using the bootstrap procedure implemented in the R-package ‘bootnet’ (1000 bootstraps; all other parameters set to default). The CS coefficient represents the maximum proportion of cases that can be dropped while maintaining a correlation of above 0.70 between the original network indices (eg, node strengths) and indices generated from subsets with the reliance on 95% probability.^23^ Following recommendations by Epskamp *et al*,^23^ we considered a CS coefficient above 0.25 to show an acceptable level of stability.

Edges connecting symptom nodes between, rather than within, family members were defined as ‘bridge edges’, and the corresponding nodes as ‘bridge nodes’. As bridge edges are the focus, networks were displayed setting non-bridge edges to zero. Bridge nodes’ centrality (bridge strength and bridge betweenness) was calculated using the R-package ‘networktools’, with default settings. Bridge strength measures a bridge node’s connectivity with those of other family members, and bridge betweenness measures the number of times a bridge node lies on the shortest path between two nodes from distinct family members.^24^ A glossary of terms used in the network analysis is provided in online supplemental table S1.

Finally, it is reasonable to assume that the structure of family mental health networks may vary across different subgroups, such as by the gender and age of children and young people, as well as household income.^25 26^ Subgroup networks were then analysed for girls and boys, children in different age groups (under 10, 10–12 and 13–15), and families in the top or lowest third of net monthly income. Differences in bridge edge strength between subgroup networks were tested using the R package ‘Network Comparison Test’ (NCT). The number of permutations was set to 1000, and multiple testing was addressed using the Bonferroni-Holm correction.^27^ All other parameters were set to their default values. The absolute difference in bridge edges was calculated (represented by ‘*E*’).

### Statistical analysis: longitudinal network on family emotional symptoms

Panel network models were fitted on nodes representing five emotional symptoms in children and four in both mothers and fathers. Data were treated as continuous, and models were estimated using the robust full-information maximum likelihood estimator using the R-package ‘psychonetrics’ and visualised with ‘qgraph’. Parameters were set to their default values unless otherwise specified in the model development procedure below. The analyses were based on complete cases. Families with fewer than two waves of data were excluded, leaving 3757 families across waves 1–13.

The longitudinal network used a graphical vector-autoregression model to capture predictive effects—or directed partial correlations—between nodes over time, with effects modelled via regression on the previous measurement occasion.^28^ Autoregressions (paths between identical nodes across waves) reflect within-family carry-over effects. Cross-lagged effects, which are paths linking two different nodes across waves, reflect the average within-family change—specifically, the extent to which changes in one symptom (eg, unhappiness in a child) are predicted by prior deviations in other symptoms (eg, unhappiness in a mother). The model also includes a random intercept representing each family’s mean structure, thus controlling for variance that results from stable trait-like family difference.

Following the suggestions of Epskamp,^28^ the final panel network was established by evaluating three nested network models. First, a baseline ‘saturated’ network model was estimated, where all edges were included. Second, a pruned model was estimated, where non-statistically significant associations (p<0.05) were recursively pruned. Finally, a stepwise-up search re-added associations with the strongest modification index until Bayesian Information Criterion (BIC) values no longer improved. Akaike Information Criterion (AIC) was also calculated, with the best-fit model having the lowest AIC and BIC values. Model fit was further assessed by the absolute and incremental fit index.^29^ For the absolute fit index, the Root Mean Square Error of Approximation was used, with values below 0.06 indicating a reasonable fit. The Bender’s Comparative Fit Index was used as an incremental fit index, with values above 0.95 indicating a good fit.

## Results

The analysis sample consisted of 4359 girls and 4436 boys (table 1). The average age was 10.96 (SD=3.21) for girls, 10.82 (SD=3.23) for boys, 40.47 (SD=6.50) for mothers and 43.28 (SD=7.34) for fathers. 

**Table 1:** Descriptive statistics of the study sample

Variable	n	%
Child gender		
Girl	4359	49.56
Boy	4436	50.44
Parents	8795	100
Age of child		
<10	2186	24.86
10–12	3340	37.98
13–15	3267	37.15
Income group*		
Higher-income households	2903	33.33
Lower-income households	2903	33.33
SDQ score of child (mean±SD)		
Internalising symptom	4.22±3.27	
Externalising behaviours	5.75±3.55	
Prosocial behaviours	2.00±1.80	
GHQ-12 score of mother (mean±SD)		
Emotional symptoms	7.76±2.58	
Social dysfunction	12.56±2.36	
Loss of confidence	3.23±1.39	
GHQ-12 score of father (mean±SD)		
Emotional symptoms	7.48±2.42	
Social dysfunction	12.36±2.11	
Loss of confidence	3.01±1.31	

* Lower income households were defined as those in the bottom third of monthly household net incomes (<2719.61 GBP) and higher socioeconomic status were those in the top third monthly household net income >4003.66 GBP).

GHQ-12, General Health Questionnaire-12; SDQ, Strengths and Difficulties Questionnaire.

### Cross-sectional network: overall family mental health

**Figure 1:**
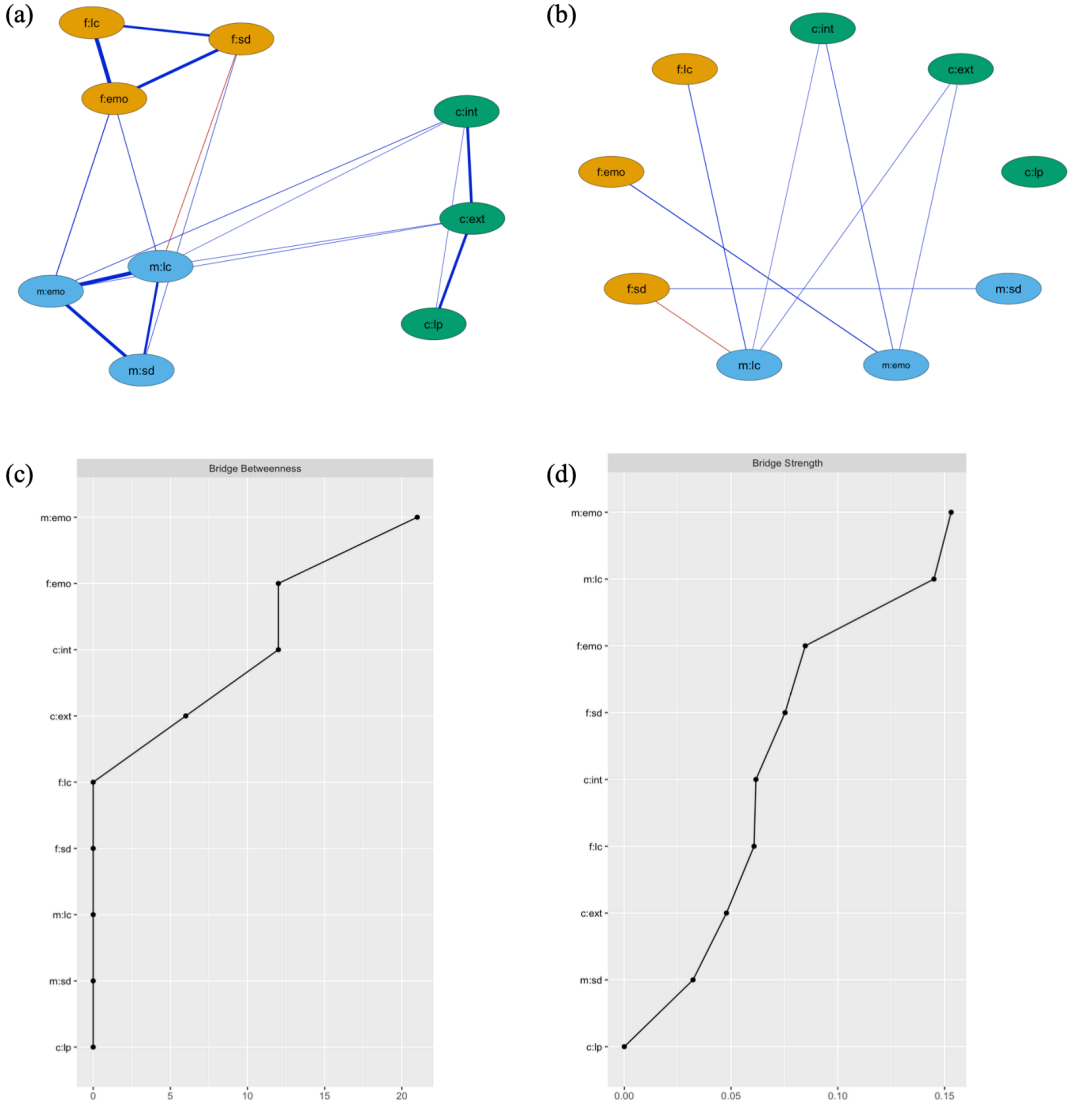
Results from cross-sectional family mental health network: (**a**) full network structure. The strength of association is represented by the length and width of line, positive and negative associations are represented by blue and red edges, respectively, (**b**) network highlighting only the bridge edges and (**c**) bridge betweenness a measure of whether a node is a key connection point between two family members, (**d**) bridge strength a measure of a family members’ mental health’s connectivity with nodes from other family members). c, children’s nodes (green); emo, emotional symptoms; ext, externalising behaviours; f, fathers’ nodes (yellow); int, internalising symptoms; lc, loss of confidence; lp, less pro-social behaviours; m, mothers’ nodes (blue); sd, social dysfunction.

In the cross-sectional network, as expected, edges (representing independent correlations) connecting mental health dimensions within the same family member were considerably stronger than those linking different family members (figure 1a). However, significant bridge edges connecting mental health dimensions between family members emerged (figure 1b). There were positive associations between maternal and paternal emotional symptoms, social dysfunction and loss of confidence. Fathers’ social dysfunction was negatively associated with maternal loss of confidence, indicating that the lower a father’s social functioning, the higher a mother’s self-confidence. Furthermore, maternal mental health nodes, including emotional symptoms and loss of confidence, were linked to increased levels of internalising and externalising symptoms in children, whereas paternal nodes were not independently related to child symptoms.

The network had good stability, as indicated by CS coefficients of 0.75 for both edge structure and strength. Maternal emotional symptoms had the largest estimated ‘bridge betweenness’ (whether a node is a key connection point between two family members; figure 1c) and ‘bridge strength’ (measure of a family members’ mental health node’s connectivity with nodes from other family members; Figure 1d). This demonstrates that maternal emotional symptoms were central in the family mental health network.

### Cross-sectional network: family mental health networks by subgroups

**Figure 2:**
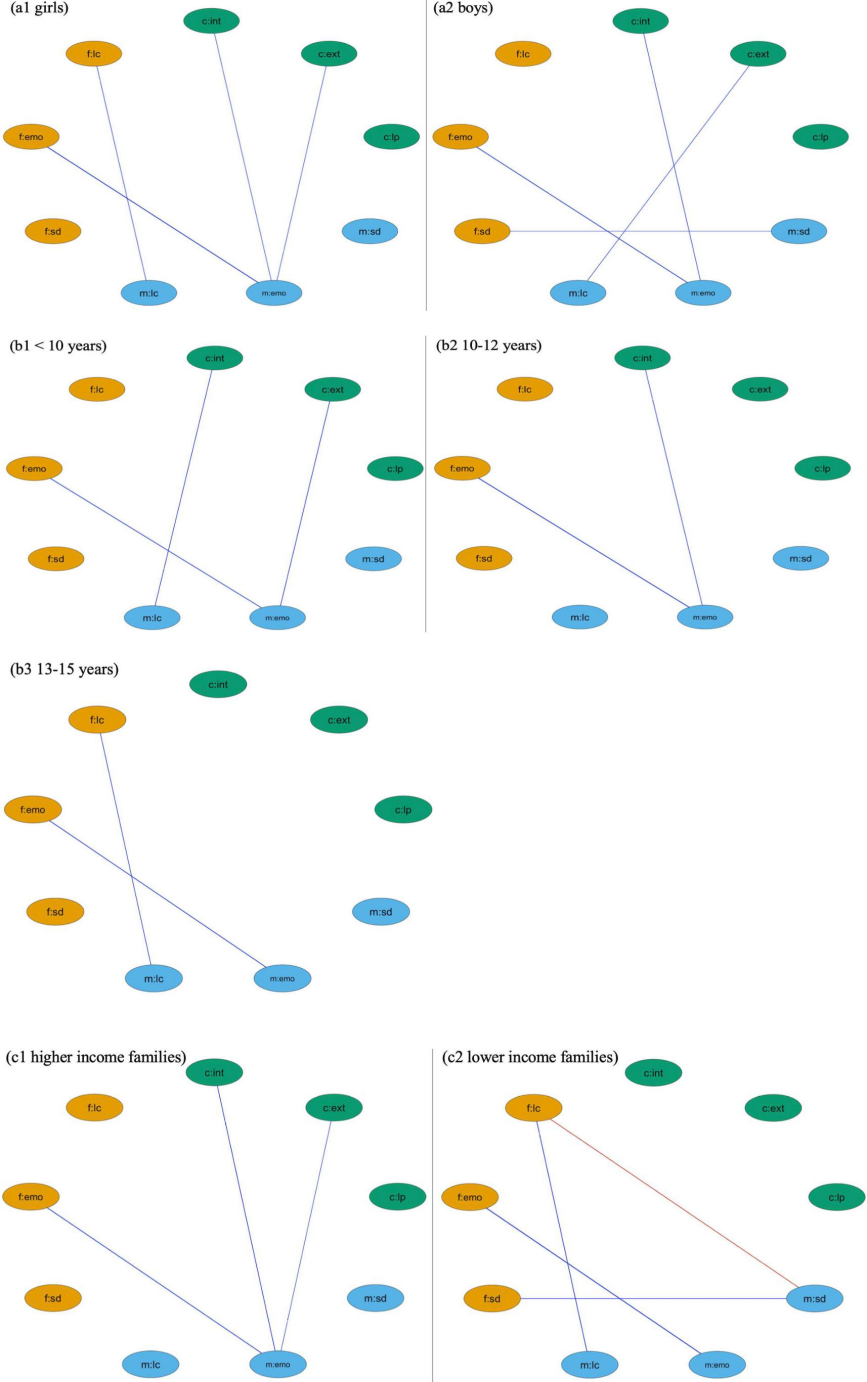
Subgroup network analysis. (**a1**) and (**a2**) network analysis for families with adolescent girls and boys; (**b1**), (**b2**) and (**b3**) network analysis for families with children (under 10 years), younger (aged 10–12) and older adolescents (aged 13–15), (**c1**) and (**c2**) network analysis for higher (top 1/3 incomes) and lower income families (bottom 1/3 incomes). c, children’s nodes; emo, emotional symptoms; ext, externalising behaviours; f, fathers’ nodes; int, internalising symptoms; lc, loss of confidence; lp, less pro-social behaviours; m, mothers’ nodes; sd, social dysfunction.

There were similarities in network structure estimated using family triads that had girls (n=4359) and boys (n=4436), such that, for both family types, fathers’ emotional symptoms were positively connected with mothers’ which, in turn, were associated with children’s increased internalising problems (figure 2, a1–a2). However, maternal emotional symptoms were also related to girls’ increased externalising problems, while maternal lower confidence levels were associated with higher levels of externalising problems among boys. Network graphics according to age group (figure 2, b1–b3) showed more edges in families with younger children compared with older ones. For children under 10 years (n=2186), two connection pathways between parental and children’s mental health were observed. This decreased to one connection for younger adolescents (aged 10–12 years, n=3340) and no connections for older adolescents (aged 13–15 years, n=3267). Similarly, there were no connections between mental health of parents and children from lower-income households (figure 2, c2, n=2903). For families from higher-income households (figure 2, c1, n=2903), paternal emotional problems were positively associated with maternal emotional problems, which in turn, were linked to higher levels of children’s internalising and externalising symptoms.

For all subgroup networks, mothers’ emotional symptoms had the highest bridge betweenness and strength, suggesting they are most implicated in the transmission pathways and exhibit the strongest connectivity compared with other nodes (see online supplemental figure S3 for a full report on bridge centrality). However, NCTs suggest that most of the observed differences in bridge edges were non-significant, except for the difference in edges connecting maternal loss of confidence and child internalising symptoms between networks for children and young adolescents (*E*=0.05, p=0.040, see online supplemental table S2 for a full report on NCTs).

### Longitudinal network for directional associations between family members’ emotional symptoms

**Figure 3:**
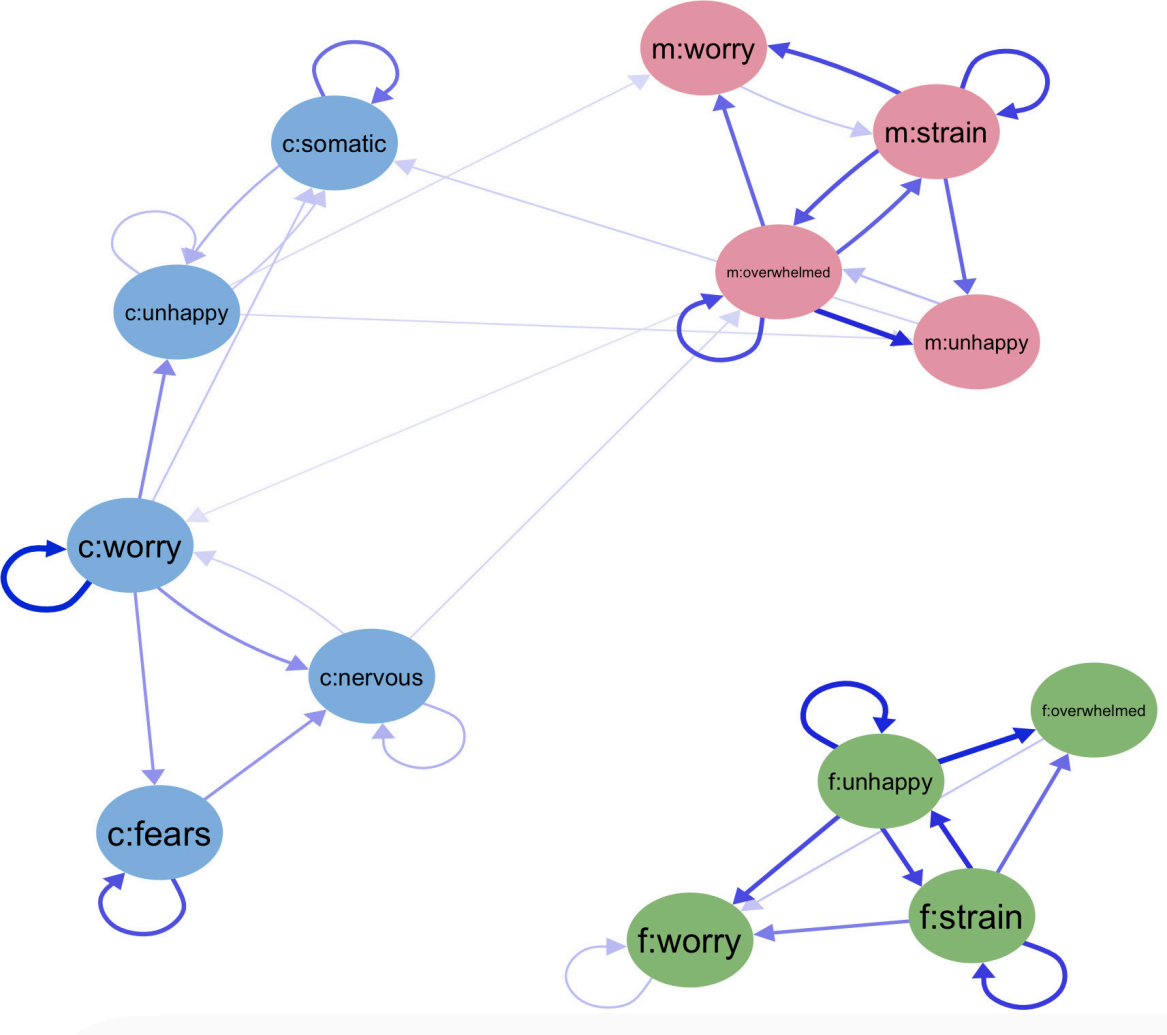
Panel network for directional temporal associations between family members’ emotional symptoms. Highly connected nodes had shorter edges and thicker association lines, and positive and negative associations were represented by blue and red edges respectively. c, children’s nodes; f, fathers’ nodes; m, mothers’ nodes.

The final longitudinal network model (figure 3, see online supplemental appendix S2 for model selection) shows that, again, paths representing temporal symptom correlations within a family member were considerably stronger than paths linking different family members. For example, an increase in a mother’s feeling of being overwhelmed predicted not only an increase in her own unhappiness at a later wave, but also a subsequent increase in the child’s worry, although the strength of the latter correlation was less strong.

Like the cross-sectional network, emotional symptoms of mothers and children were linked, while changes to a father’s emotional symptoms were not independently related to future child’s or the mother’s symptoms, nor vice versa (figure 3). Being overwhelmed and strained appeared to be central difficulties for mothers, as these symptom sets influenced all of their three other emotional symptoms. The mother’s increased feeling of being overwhelmed predicted an increase in the child’s worry at a later wave. The child’s worry, in turn, contributed to the development of the child’s other symptoms (nervousness, unhappiness, fears and somatic symptoms). The increased level of nervousness in the child, in turn, fed into the mother’s feeling of being overwhelmed, while the child’s unhappiness contributed to the development of the mother’s unhappiness and worry at a later wave. Therefore, there seems to be a transmission pathway that spreads from mothers to children and then back to mothers: mother’s feeling overwhelmed contributed to children’s worry, which was then associated with increased nervousness and unhappiness in children, ultimately feeding into the mother’s emotional symptoms, leading her to feel more overwhelmed, worried and unhappy.

## Discussion

Several key findings emerged when we used cross-sectional and longitudinal network approaches to capture how family members influence each other’s mental health. First, we confirm that the mental health of mothers plays a central role in the family mental health network, particularly maternal emotional well-being. Second, the longitudinal analysis reveals a feedback loop in emotional symptoms between mothers and children, centred around anxiety symptoms. A mother’s feelings of being overwhelmed not only affected the child’s emotional state—especially worry—but also cycled back, further affecting her own emotional health (eg, increasing her worry and feeling overwhelmed). Lastly, while fathers were linked to a mother’s mental health in the cross-sectional analysis, we observed a notable absence of independent associations between fathers’ emotional symptoms and those of their children.

The finding that maternal mental health is more strongly linked to their children’s mental health than the fathers’ aligns with a wealth of prior knowledge linking maternal effects more strongly than paternal effects to child outcomes.^30^ However, the present finding is not consistent with some work which reports associations between paternal emotional distress and children’s mental health, after accounting for maternal mental health.^31^ This divergence may arise because, when multiple mental health domains are modelled simultaneously, maternal mental health may mediate most of the effect of paternal mental health on children’s well-being. For example, fathers’ emotional symptoms may influence children’s well-being indirectly by affecting maternal mental health. Indeed, in the cross-sectional network, we observed associations between each of the mothers’ and fathers’ mental health domains, underscoring the relevance of paternal mental health within the family mental health system. Another network study examining depression symptoms in parents reported cross-sectional links between fathers and mothers^12^; we do not report such maternal-paternal links between emotional symptoms in our longitudinal study. This suggests that much of the observed association is because of non-causal confounders, such as shared socioeconomic circumstances and assortative mating.^32^

There are several reasons why maternal mental health may be more strongly related to their children’s than a fathers’. One key consideration is the traditional division of parenting roles, where societal expectations often position mothers as the primary caregivers and organisers within families. As a result, mothers are typically more involved in child-rearing activities and spend considerably more time with their children than do fathers, which can provide more scope for maternal mental health to influence children’s lives and development. More recent literature suggests that mothers play a primary role in supporting children’s emotional functioning, while fathers tend to act as loving playmates.^33^ From a social learning perspective, greater exposure to maternal care might lead children to model their mother’s coping mechanisms and behaviours. It is, therefore, possible that, if a parent is struggling with poor mental health, children are more likely to encounter emotional distress and maladaptive parenting behaviours in mothers than in fathers.^30^ Moreover, the environments mothers navigate are often more challenging. Women are more likely to face societal disadvantages such as intimate partner violence, low paid work, single parenting, poverty and discrimination.^34^ Such factors are known to harm children’s development. Lastly, the current sample contains non-biological parents. It is reasonable to assume that, in such ‘blended families’, the father-child association may be weaker for stepchildren: there is a higher likelihood for children of separated parents to live with the mother and stepfather.^35^ Indeed, in our sample, 88.5% of children in blended families lived with their biological mother.

In our findings, maternal emotional health—particularly anxiety symptoms—is central to family mental health. Mothers’ experiences of feeling overwhelmed and under strain led children to experience worry in subsequent waves, which, in turn, activated children’s symptoms of anxiety (eg, nervousness and fear) and depression (eg, unhappiness). Although feelings of being overwhelmed and strained could be seen as symptoms of anxiety^36^ or as consequences of other disorders (eg, depression or functional impairment), the direction of the associations from our panel network analysis suggests these feelings function primarily as originating symptoms rather than arising from other symptoms measured in the GHQ-12. For example, maternal feelings of overwhelm strongly predicted later depressive symptoms (eg, unhappiness), with a much stronger estimated association in this direction than in the reverse. Therefore, while much research has focused on maternal depression, our findings highlight the critical role of maternal anxiety and stress in shaping both maternal and child mental health. Overwhelming stress can reduce a mother’s capacity to provide care and support for her children.^37^ Anxious mothers may engage in parenting behaviours characterised by high levels of control (eg, overprotection) and lower levels of parental care, which can adversely affect children’s emotional well-being.^38^ Our results also indicate that children’s emotional symptoms, particularly their unhappiness, fed into maternal stress, leading mothers to be more worried, overwhelmed and unhappy. Mothers of children with mental health difficulties may also, in turn, face greater demands and feelings of guilt over perceived parenting effectiveness, which can result in reduced parental satisfaction, lower confidence and heightened emotional distress.^39^

Subgroup analyses show that maternal emotional distress exhibited stronger associations with higher symptoms in younger children, girls and in families from higher socioeconomic status. Although global comparisons between subgroup’s networks were largely non-significant, these patterns potentially reflect different family mental health dynamics. For instance, as children grew up, the influence of maternal emotional health on their children diminished. This may reflect how adolescents transfer their primary attachment figures from their parents/caregivers to peers and/or romantic partners.^40^ In the present study, lower levels of maternal confidence showed a significantly stronger association with internalising symptoms in younger children (<10 years) compared with early adolescents (10–12 years). This may be because, in the current sample, the mental health of younger children was reported by their caregivers, who, in most cases, were their mothers, raising the possibility of shared method variance bias.

### Clinical implications

These results raise important implications for clinical practice and public health. First, our findings suggest that emotional symptoms of mothers are more strongly linked within a family system than other dimensions of mental health. Among emotional symptoms, anxiety symptoms, such as a mother feeling overwhelmed and strained, are central in the transmission pathway. This highlights the potential value of interventions that aim to support mothers and reduce maternal anxiety, which may have the greatest impact on improving family dynamics and reducing the risk of poor mental health in children.

However, it is crucial to situate these findings within a broader societal context. A mother becoming overwhelmed is likely to reflect structural inequalities for women, perhaps especially when they become parents, compared with those experienced by men and fathers. For example, it is well-documented that when women become mothers, they face disproportionate pressures from both workplace and family environments.^41^ It is perhaps surprising then that a recent systematic review identified no randomised controlled trials examining the effect of treatments for parental anxiety on risk of anxiety in children.^42^ This represents a significant missed opportunity to protect children from the intergenerational transmission of mental distress and to improve family mental health and that of society overall.

Our findings also suggest that paternal mental health is linked to maternal confidence and social functioning, presenting a further potential avenue for alleviating maternal stress. A recent study from Sweden (which incidentally has the highest uptake of parental leave among fathers globally), for example, suggests that paternal leave following birth reduced mother’s mental health-related service use and prescriptions for anti-anxiety drugs.^43^

### Limitations

There are several limitations to the current study. First, although multiple mental health dimensions were captured for parents, externalising behaviours were not included. Second, we included family structures where a triad of children and two parents was present, which accounted for 75.6% of households with children in UKHLS. It is likely that the availability of complete triadic data is socioeconomically patterned. Previous research has demonstrated phenotypic differences between children from complete trios versus duos (ie, missing one parent), with trios showing lower levels of psychopathology and conduct disorder symptoms.^44^ This selective inclusion could result in a sample that is not fully representative of the broader population. The longitudinal network analysis included a smaller number of families, due to sample attrition and the exclusion of older children who aged out of the youth sample across waves. This reduced sample size may have limited the statistical power to detect associations and introduced additional bias if attrition was non-random. Future studies should extend the approach by including a wider range of family structures.

Another limitation was that in the analysis, we treated the Likert items as approximately continuous and modelled them under a Gaussian assumption, partially due to software availability, as treatments of ordinal data in network approaches remain suboptimal.^45^ Treating Likert-scale data as continuous is common in psychometric modelling,^21 46^ and Gaussian methods have been shown to be relatively robust to violations of normality when the number of categories is small.^22 47^ Nonetheless, this approach assumes equal spacing between response options and may bias parameter estimates. Alternative semiparametric approaches, such as nonparametric extensions of undirected graphical models,^48^ have been proposed to relax the normality assumption when estimating undirected graphs. However, their wider uptake in applied research is likely to depend on the availability of more accessible, user-friendly software.

Moreover, this study adopted a data-driven approach and did not control for demographic or socioeconomic covariates. While this aligned with our aim to characterise the overall structure of mental health networks in families, some of the associations could arise due to their shared relationship with confounders that future hypothesis-driven research should address. We acknowledge unmeasured risk factors. For example, we included non-biological families. We acknowledge the effects of unmeasured risk factors in stepfamilies such as parental separation and biological father’s mental health conditions. Also, the network analysis does not account for the role of genetic transmission in parent-offspring associations. Finally, even though we modelled family emotional symptoms network using a longitudinal approach, we estimated undirected associations among family members’ mental health dimensions. As a result, we are unable to determine the directionality of these connections. Future analyses should employ designs with greater statistical power to better ascertain the direction of effects across different mental health domains.

### Conclusion

By characterising the family, more realistically, as a network of individual family members’ mental health symptoms nested within a family system, we have demonstrated more clearly how family members influence each other’s mental health simultaneously. This has allowed us to confirm the stronger maternal effects and untangle the directional transmission pathway of emotional symptoms over time within the family unit. Maternal anxiety emerges as a key target for preventing the transmission of mental distress within families and promoting young people’s mental health. Future research should identify the optimal timing and nature of interventions for families and clarify the additional benefits of targeting maternal mental health for improving child outcomes.

## Supplementary material

10.1136/bmjopen-2025-104829 online supplemental file 1

## Data Availability

Data are available in a public, open access repository.
