# Supplementary material for: Quantifying cross-sectional and longitudinal associations in mental health symptoms within families: network models applied to UK cohort data
Source: BMJ Open. 2025 Oct 6;15(10):e104829. doi: 10.1136/bmjopen-2025-104829 (PMC12506059; doi:10.1136/bmjopen-2025-104829)
Supplement: online supplemental file 1 [file bmjopen-15-10-s001.docx]

**Supplementary Materials for: Understanding family mental health using cross-sectional and longitudinal network models**

**Supplementary Appendix S1: Cross-sectional family mental health network based on one child per family**

As noted in the main text, we conducted a sensitivity analysis by randomly selecting only one child per family for the cross-sectional network models. This resulted in a sample of 7,185 families. While fewer associations were detected compared to the main analysis, likely due to the smaller sample size, the results were broadly consistent. As shown in Figure S1, edges connecting mental health dimensions within the same family member were considerably stronger than those linking different members. Nonetheless, significant bridge edges between family members’ mental health dimensions also emerged. Consistent with the main findings, maternal emotional symptoms were again associated with increased internalising and externalising symptoms in children, whereas paternal symptoms were not independently linked to child outcomes.

**
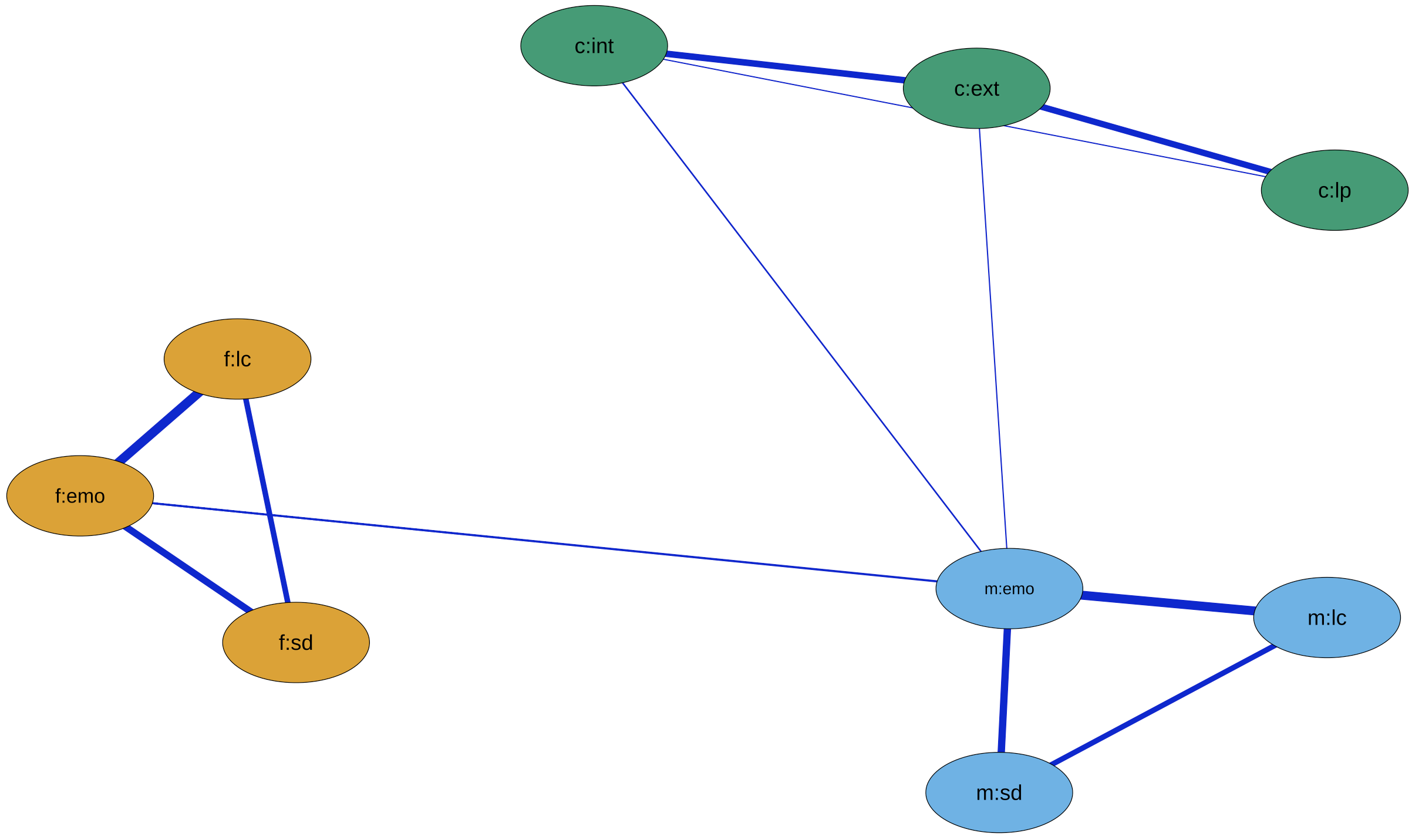
**

Figure S1 Cross-sectional family mental health network based on one child per family. The strength of association is represented by the length and width of line, positive and negative associations are represented by blue and red edges respectively. m = mothers’ nodes (blue); f = fathers’ nodes (yellow); c = children’s nodes (green); emo = emotional symptoms; sd = social dysfunction; lc = loss of confidence; int = internalising symptoms; ext = externalising behaviours; lp = less pro-social behaviours

**Figure S2. Pairwise scatterplots, histograms, and correlation coefficients among mental health dimensions from children, mothers, and fathers.**

**
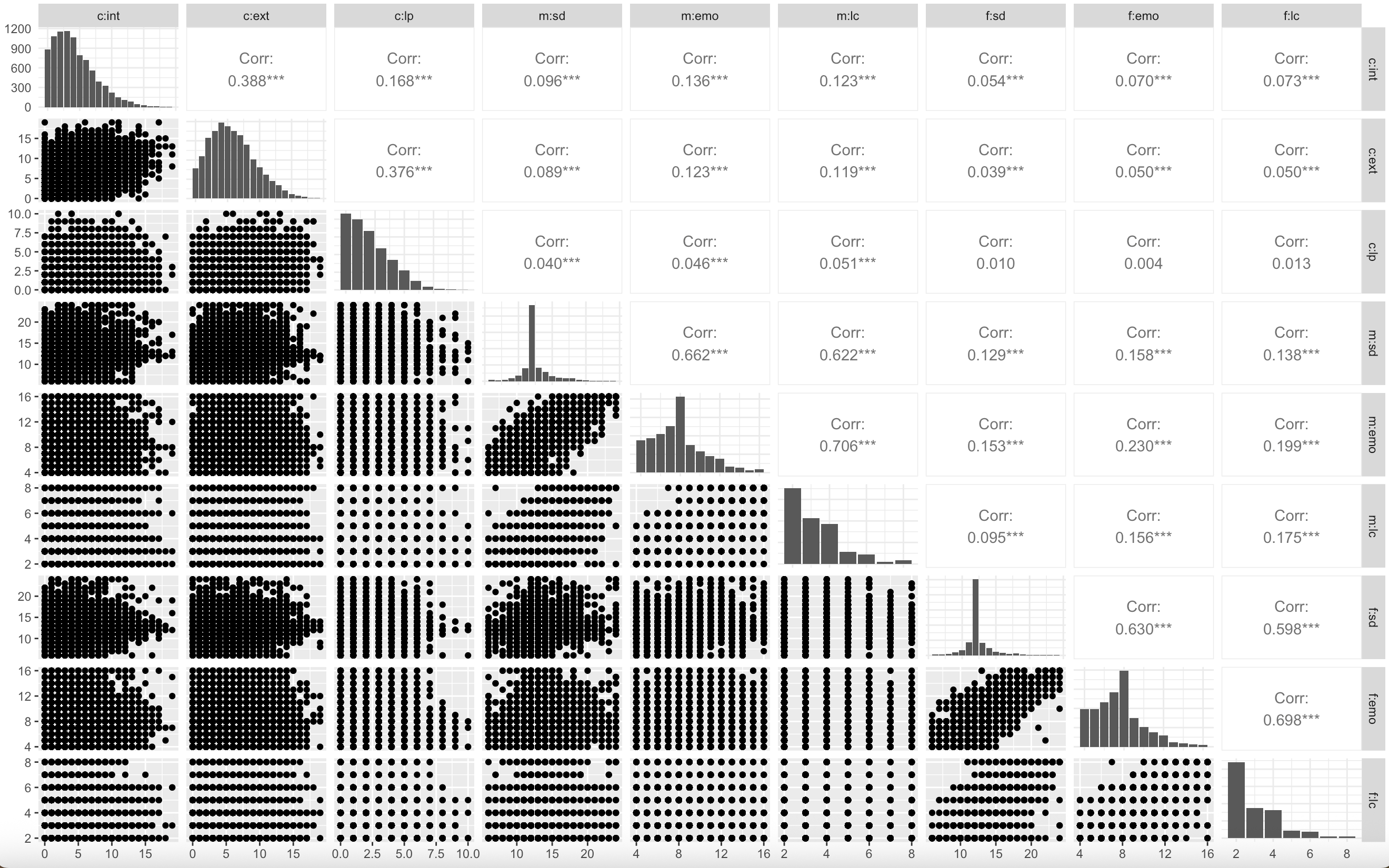
**

Note. The plots were generated using the ggpairs function from the GGally R package. The bottom-left panel displays pairwise scatterplots illustrating bivariate relationships between variables. The diagonal panel shows histograms of the marginal distributions for each variable. The top-right panel presents Pearson correlation coefficients for each variable pair.

m = mothers’ nodes; f = fathers’ nodes; c = children’s nodes; emo = emotional symptoms; sd = social dysfunction; lc = loss of confidence; int = internalising symptoms; ext = externalising behaviours; lp = less pro-social behaviours

**Table S1 Glossary of Terms**

| Key terms | Descriptions |
| --- | --- |
| Nodes | Family members mental health domains |
| Edges | Independent correlations between nodes in the network |
| Bridge edges | Edges connecting two family members, rather than edges connecting mental health symptoms within an individual |
| Bridge nodes | Nodes associated with bridge edges |
| Bridge strength | The total strength of a node connecting nodes from other family members. It identifies family member's mental health dimensions that are most strongly connected with other family member’s mental health. |
| Bridge betweenness | The number of times a node lies on the shortest path between two nodes from distinct family members. It identifies family member’s mental health domains most implicated in the transmission of mental health within a family. |

**Figure S3. Bridge centrality statistics for networks estimated on subgroups according to children's gender, age, and family socioeconomic status**

Note. Bridge centrality includes bridge betweenness (whether a node is a key connection point between two family members) and bridge strength (measure of a family members’ mental health’s connectivity with nodes from other family members).

m = mothers’ nodes; f = fathers’ nodes; c = children’s nodes; emo = emotional symptoms; sd = social dysfunction; lc = loss of confidence; int = internalising symptoms; ext = externalising behaviours; lp = less pro-social behaviours

1. The bridge centrality for networks for adolescent girls


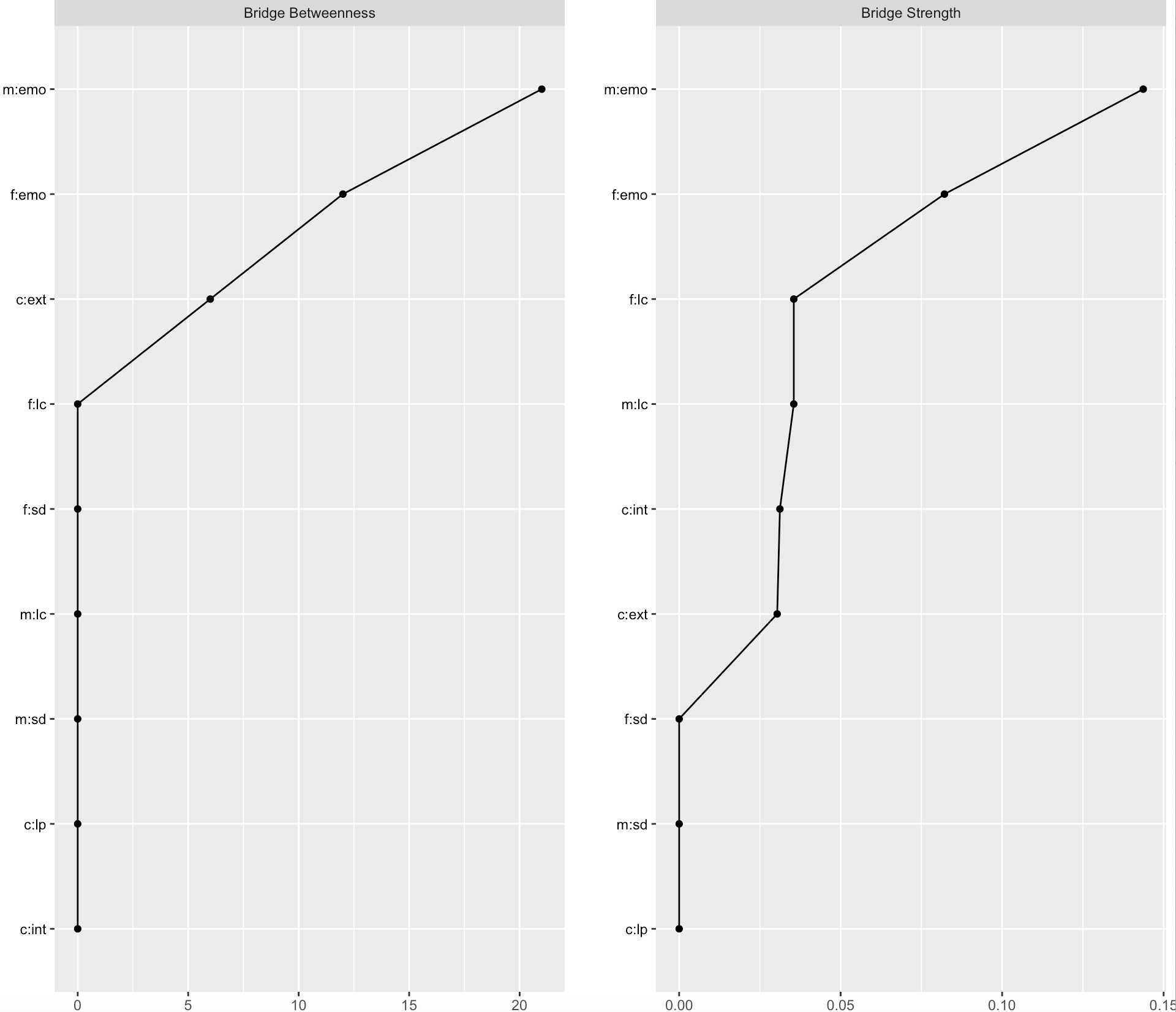


1. The bridge centrality for networks for adolescent boys


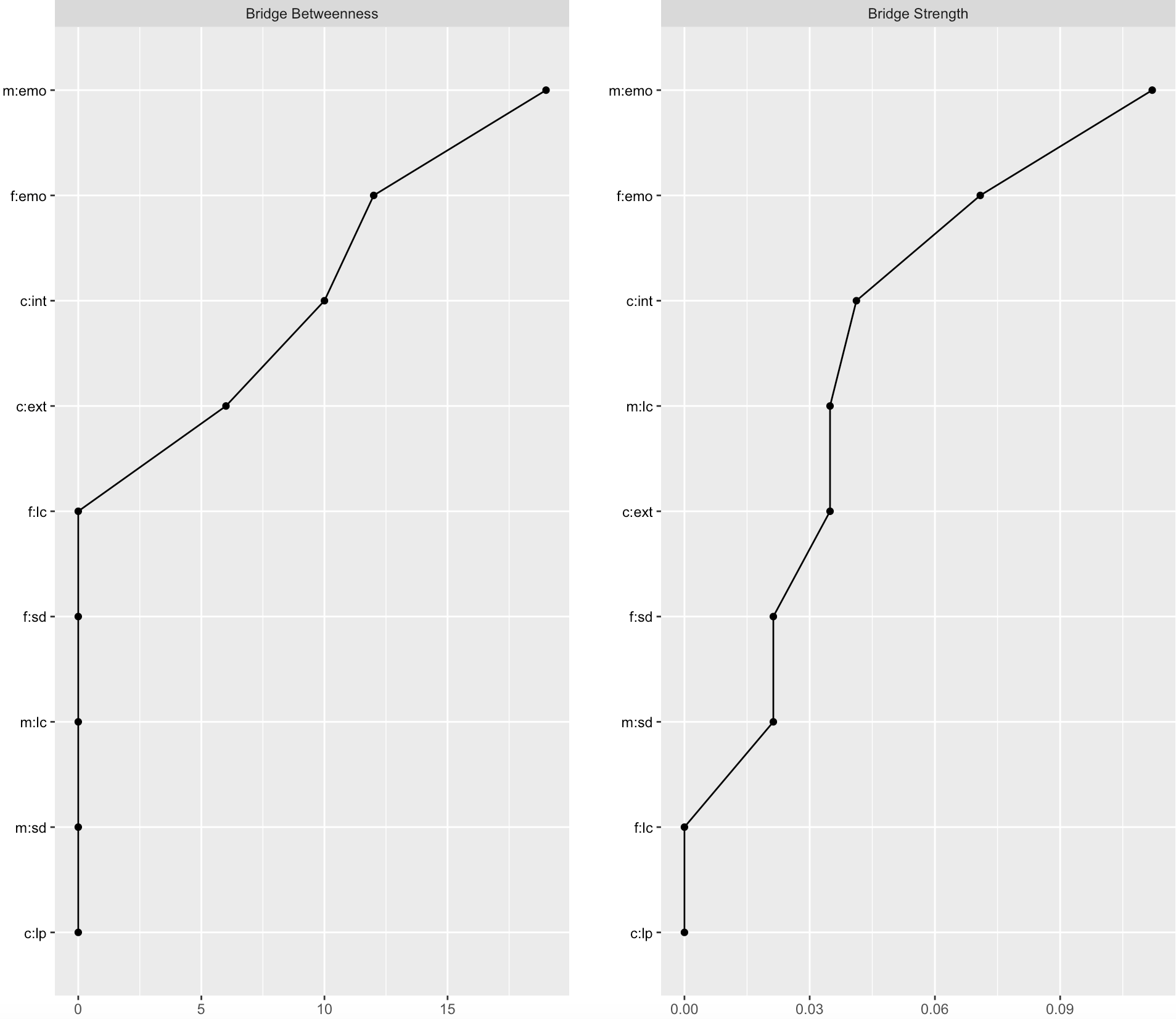


1. The bridge centrality for networks for children aged under 10 years


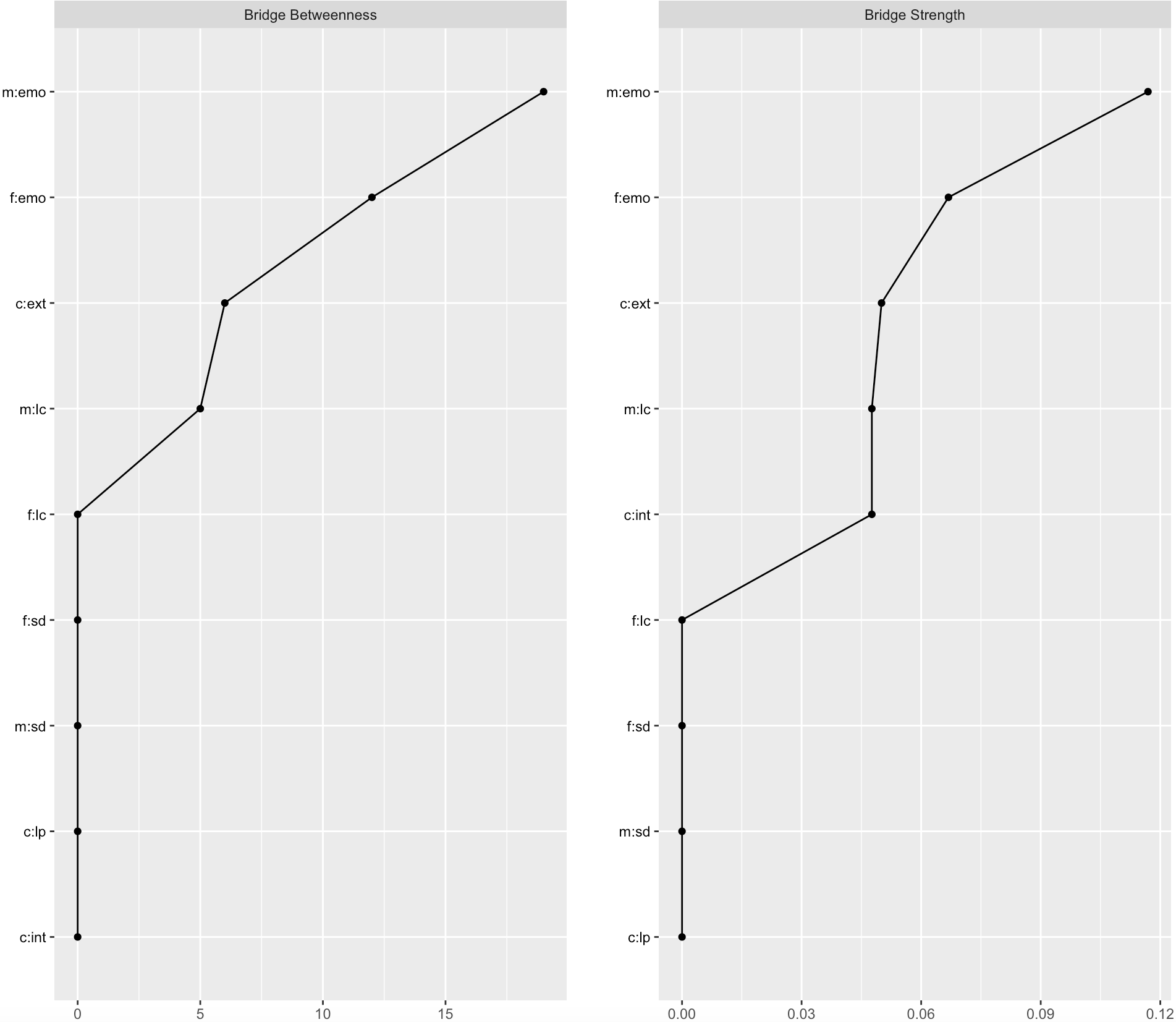


1. The bridge centrality for networks for younger adolescents aged 10-12 years


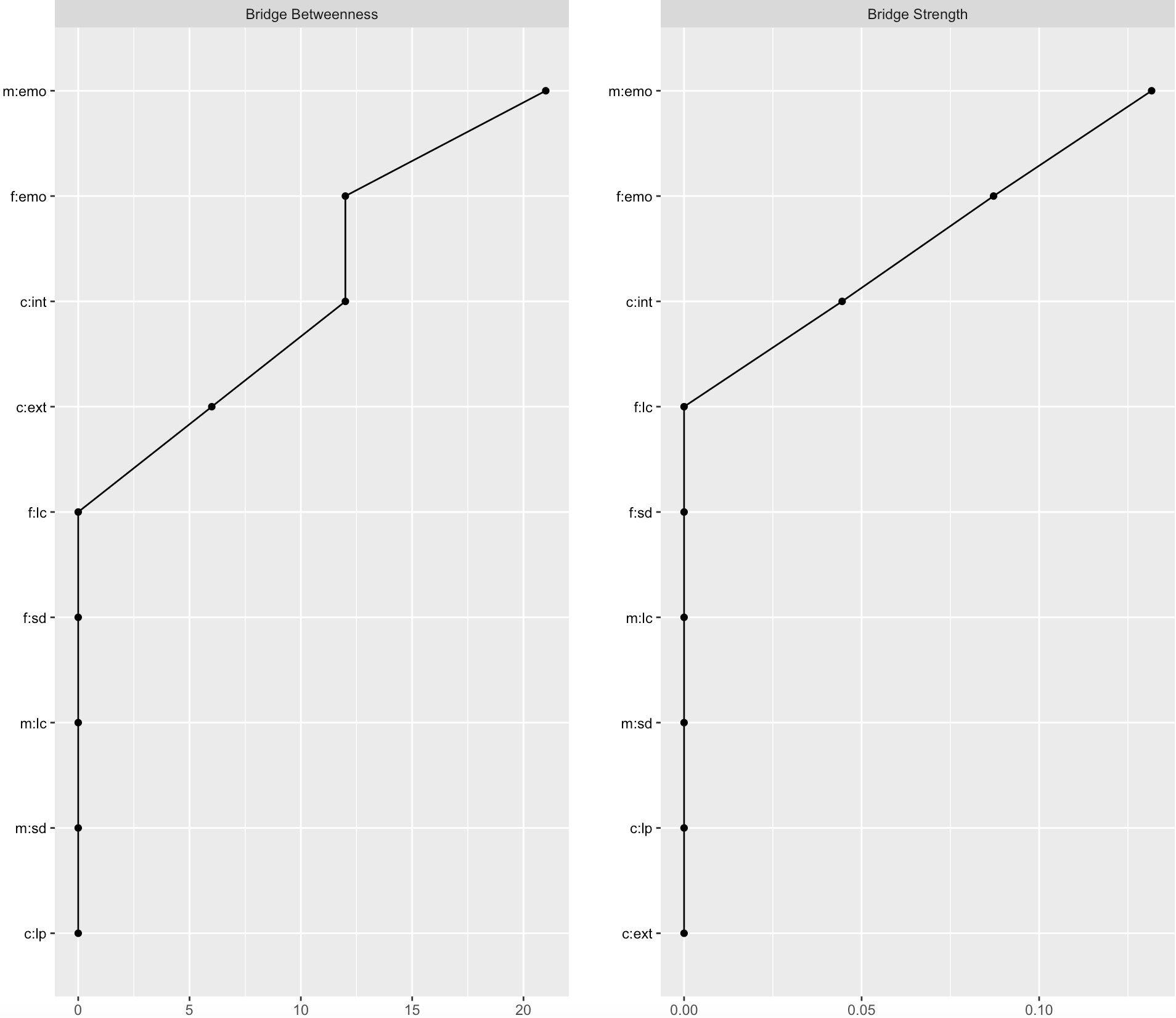


1. The bridge centrality for networks for higher income families


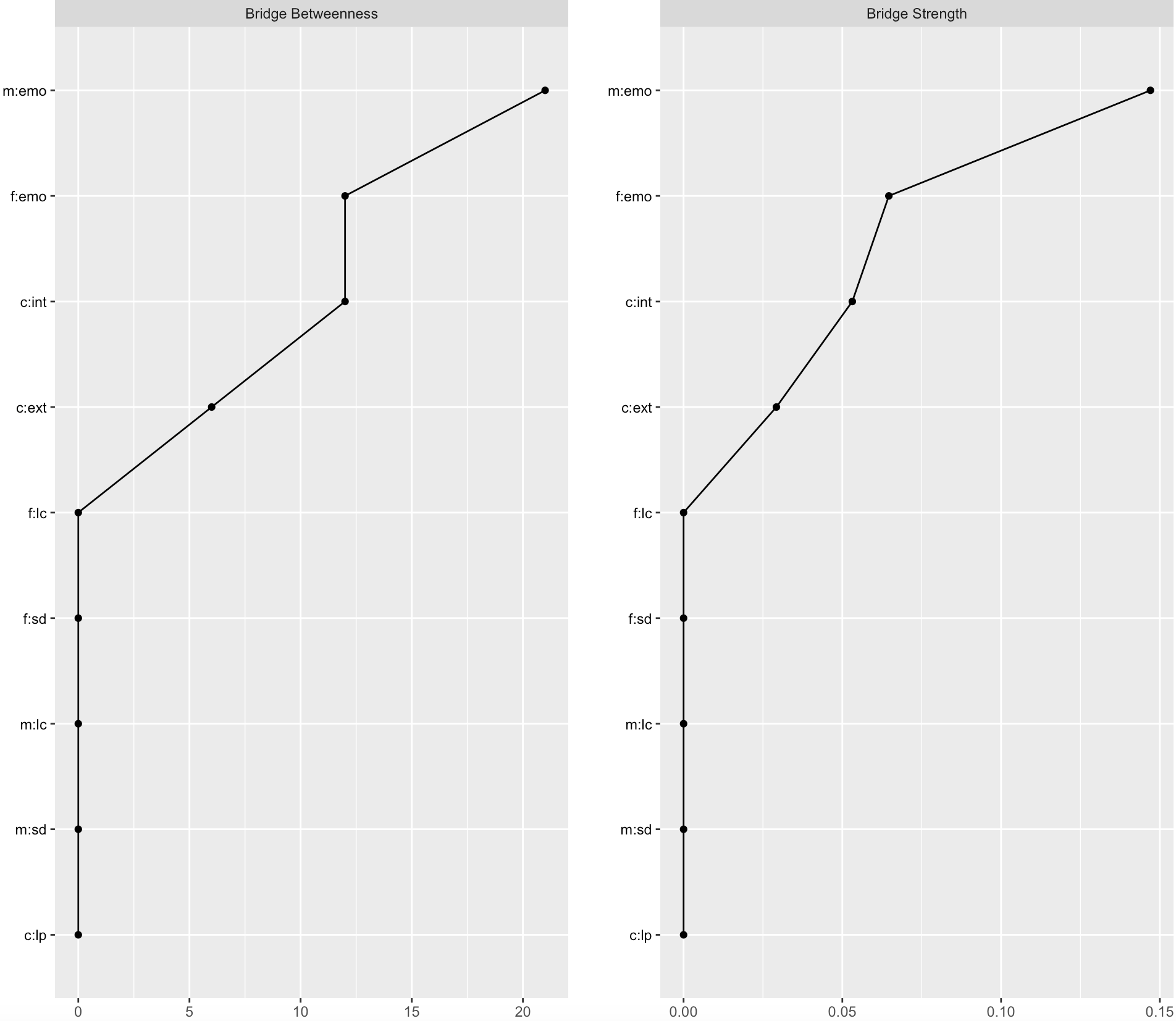


**Table S2 Results from network comparison tests comparing the strength of bridge edges’ between networks defined by subgroups**

| Subgroup comparison | | | | | | | | | | | |
| --- | --- | --- | --- | --- | --- | --- | --- | --- | --- | --- | --- |
|  | girls vs boys | |  | children vs younger adolescents | |  | younger vs older adolescents | |  | higher vs lower income families | |
| Bridge edge | *E** | *p* |  | *E** | *p* |  | *E* | *p* |  | *E* | *p* |
| m:emo - c:int | 0.01 | 1.00 |  | 0.04 | .56 |  | 0.04 | .34 |  | 0.05 | .70 |
| m:emo - c:ext | 0.03 | 1.00 |  | 0.05 | .07 |  | — | — |  | 0.03 | .82 |
| m:emo - f:emo | 0.01 | 1.0 |  | 0.02 | .56 |  | 0.03 | .34 |  | 0.02 | .82 |
| m:lc - c:int | — | — |  | 0.05 | .04 |  | — | — |  | — | — |
| m:lc - c:ext | 0.03 | .54 |  | — | — |  | — | — |  | — | — |
| m:lc - f:lc | 0.04 | 1 |  | — | — |  | 0.04 | .25 |  | 0.06 | .51 |
| m:sd - f:sd | 0.02 | 1 |  | — | — |  | — | — |  | 0.05 | .82 |
| m:sd - f:cl | — | — |  | — | — |  | — | — |  | 0.05 | .06 |

‘*E*’ represents the difference in the absolute strength of paired bridge edges between two networks; ‘*p*’ represents *p*-values that determine the significance of the difference tests.

m = mothers’ nodes; f = fathers’ nodes; c = children’s nodes; emo = emotional symptoms, including anxiety and depression; int = internalising symptoms; ext = externalising behaviours; lc = loss of confidence; sd = social dysfunction

**Supplementary Appendix S2: Model selection for the final longitudinal network model**

As mentioned in the main text, the final panel network was established by evaluating three nested network models: a baseline ‘saturated’ network model, a pruned model, and a step-up model. The fit of each model is summarised in Table S3 below. The pruned model was initially rejected due to its poor fit, as reflected by a lower CFI value (CFI = 0.90). Both the baseline and step-up models demonstrated reasonable fit, indicated by RMSEA values (< 0.06) and CFI values (> 0.95). Since the step-up model had the lowest AIC and BIC, it was selected as the final model.

Table S3 Model fit of the three nested models

| Model Type | *χ2* (*df*) | RMSEA | CFI | AIC | BIC |
| --- | --- | --- | --- | --- | --- |
| Baseline | 5116.31 (3913) | 0.01 | 0.97 | 245388.65 | 247656.87 |
| Pruned (α = 0.05) | 8096.81 (4186) | 0.02 | 0.90 | 247823.16 | 248390.21 |
| Stepup | 6324.30 (4167) | 0.01 | 0.95 | 246088.64 | 246774.09 |

RMSEA = Root Mean Square Error of Approximation, CFI = Comparative Fit Index, AIC = Akaike Information Criterion (AIC), and BIC = Bayesian Information Criterion
