# Supplementary material for: Quantifying cross-sectional and longitudinal associations in mental health symptoms within families: network models applied to UK cohort data
Source: BMJ Open. 2025 Oct 6;15(10):e104829. doi: 10.1136/bmjopen-2025-104829 (PMC12506059; doi:10.1136/bmjopen-2025-104829)
Supplement: Supplementary file 2 [file bmjopen-2025-104829.reviewer_comments.pdf]

PEER REVIEW HISTORY

BMJ Open publishes all reviews undertaken for accepted manuscripts. Reviewers are asked to complete a checklist review form and are provided with free text boxes to elaborate on their assessment. These free text comments are reproduced below.

ARTICLE DETAILS

Title (Provisional)

Quantifying cross-sectional and longitudinal associations in mental health symptoms within families: network models applied to UK cohort data

Authors

Bai, Yushi; Rayner, Archie; Abel, Kathryn Mary; Cartwright-Hatton, Sam; Wan, Ming Wai; Pierce, Matthias

---

VERSION 1 - REVIEW

**Reviewer**	**1**
**Name**	**Bottolo, Leonardo**
**Affiliation**	**MRC Biostats**
**Date**	**08-Jun-2025**
**COI**	**None**

The Authors analyse mental health data in families by using cross-sectional and longitudinal graphical models implemented in two R software packages.

I have a few comments regarding the statistical aspects of the manuscript.

Major points:

i) P(age)8:l(ine)23 - The Authors claim that a dimensional reduction of the original data set is necessary "to avoid a model with too many variables". They aggregate several SDQ items together based on previous studies. GHQ-12 items are also aggregated.

There are not many details regarding SDQ scale but GHQ-12 is a "a 12-item instrument with a 4-point response scale". If I understood correctly, GHQ-12 scale should range between 0 and 48.

Could the Authors clarify why they have chosen a Poisson link function (P9:l11) for the marginal distribution of each category made of aggregated items in GHQ-12? The marginal distribution (as well as in SDQ) should be instead a Binomial distribution given the bounded range of the scale. For instance, “loss of confidence range” ranges between 0 and 8.

From a theoretical viewpoint, approximating Binomial(n, p) with Poisson is valid if n is large and np is small. However, taking “loss of confidence range” category as an example, n = 8 and the probability of success p should be extremely small for the validity of the approximation. Is this assumption on p correct in the real data application?

From a practical viewpoint, it would be interesting to visualise the marginal distribution of the categories and all pairs correlation (for instance by using the function ggpairs in *GGally* R package). Moreover, a QQ plot of the theoretical quantiles of a Poisson distribution versus the empirical quantiles should clarify the validity of the Poisson approximation.

In the longitudinal network analysis (P10:l12), what is the marginal distribution of the “family emotional symptoms” random variables?

ii) P9:l7 - “Data from all available waves were used, with single observations randomly selected for families with repeated measures”.

If I understood correctly, all data from all available waves were used in the cross-sectional analysis. However, per family, observations from all available waves are not independent (their temporal dependencies are modelled subsequently by using a vector autoregressive model), so graphical models which assume independence of the observations cannot be applied straightforwardly. The Authors recognised this problem when they sampled at random one observation from families with repeated measures.

Can the Authors clarify this point regarding the non-independence of the observations? Is there any way to account for the random effects produced by the longitudinal nature of the data and the presence of repeated measures? Would it have been better to analyse the data only at wave 1 and use it as a baseline/reference graphical model?

iii) P9:l57 - The Authors stratify the data in the cross-sectional analysis by sex and age of the children, and family incomes. However, did the Authors correct for these covariates in the overall cross-sectional graphical model analysis? These covariates can produce spurious correlations and misleading results between the categories if not accounted for.

Are in the data set other covariates besides sex and age of the children, and family incomes that should be considered?

Moreover, when sex is used to stratify the data set, are the effects of age of the children and family incomes removed? Similarly when age of the children categories are used for the stratification.

In the longitudinal network analysis (P10:l12), family incomes covariate presumably changes over waves/time. How should it be treated to remove its effect?

Minor points:

P(age)4:l(ine)15: Please remove the term “causal”. Unless the Authors claim Granger causality, graphical models alone cannot establish causal dependency relations between time series.

P9:l11: In the cross-sectional analysis, please provide more information regarding the *mgm* R package function used and its parametrisation (if different from the default values) as well as for the *bootnet* and *networktools* packages employed in the same analysis.

P9:l5: It is unclear what the Authors mean by “independent correlations between nodes”. Do they mean a correlation graph?

P9:l11: In the longitudinal analysis, please provide more information regarding which *psychonetrics* R package function has been used and its parametrisation (if different from the default values).

**Reviewer**	**2**
**Name**	**Rice, Frances**

Affiliation **Cardiff University Division of Psychological Medicine and Clinical Neurosciences**

Date **09-Jun-2025**

COI **None**

This is an interesting paper that describes analysis family based network analysis from the Understanding Society cohort. It analyses data from mothers, fathers and children in order to better understand how symptoms of mental health problems in family members influence each other. While the paper makes an interesting read and generates some novel findings there are some aspects of the work that could be revised to improve the paper. Specifically, there are some points of clarification, some queries/suggestions about how the study is set-up in the introduction and, most importantly, some aspects of the discussion that should be toned down slightly.

Introduction and discussion

Quite a bit of the rationale for the study outlined in the introduction is based on the identification of intervention targets and a broad range of intervention studies are mentioned. Some of this rationale seems slightly over-stated because: many intervention targets for emotional problems are already known e.g. family therapy, CBT for depression in children. Moreover, some of the evidence on P4 refers to interventions for very young children in the section where it is highlighted that interventions that involve parents may be more effective. Relatively few studies are cited here and it's also not clear to what extent this applies to the full age range examined in the study. Please consider if these statements are needed in the establishing the rationale for the study. These points seemmore like discussion points.

P4 – the authors say "with the family serving as a key instrument of change." This statement is not justified. Please either rephrase or justify with a citation.

P4 – an incomplete argument "However, to optimize……". Please state which aspects of mental health and family relationship are most important for what.

In the introduction there is a relatively long section discussing the role of genetic influences in intergenerational transmission (P5). This seems misplaced because this study cannot control for genetic influences. Also, the range of potential genetic influences at play is not considered such as shared genes but also processes such as passive gene environment correlation. Finally inter-generational processes differ for externalising and internalizing problems. This latter point suggests that the intervention targets are likely to be different for different aspects of mental health. Please consider whether this section is needed in the introduction. This seems more like a discussion point.

The authors may wish to mention family systems theory in introduction.

P5 the authors say ….”this makes it difficult to disentangle the specific…..” That is true but it’s not clear how the present study addresses that. It’s therefore not clear if this statement is needed.

The rationale for focusing on emotional problems seems a little odd because it’s a rationale for focusing on emotional problems completely. Whereas one analysis focuses on emotional problems (longitudinal) and one does not. Perhaps re-think the justification?

Missing details

Are multiple children per family included?

If yes, how is the non-independence accounted for.

Please describe the GHQ in more detail – what is it thought to measure P7.

There is no explanation or justification of the subgroup analyses on P8. Please add to P8.

How is missing data handled in the longitudinal analysis.

Queries

The authors interpret the finding from the symptom of being overwhelmed as being driven by anxiety. I am not sure that feeling overwhelmed is an anxiety symptom as per discussion. It could be a symptom of anxiety, or it could driven by hopelessness or functional impairment. I would suggest broadening the focus in the discussion of this result i.e. it’s hard to justify that it’s only anxiety.

P14. The authors say that “women tend to experience more enduring mental health problems than fathers”. I’m not sure this is justified. Please justify or rephrase.

On P14 the authors say that the association between fathers and children may be weaker because it includes step-children. Are the authors able to provide some detail on this e.g. are they able to report the likely % of step-father families or test this?

Discussion and limitations

An important limitation not mentioned is that only 75% of the sample with children in Understanding Society met the inclusion criteria. It seems likely that there is socio-economic patterning of complete trio data (e.g. other studies have shown ascertainment bias for complete father, mother and child trios when collecting DNA samples e.g. West https://pubmed.ncbi.nlm.nih.gov/12399949/). Please mention as a limitation.

The longitudinal data set sample size is less than half that of the cross sectional sample size. This should be mentioned as a limitation. As does attrition and selective attrition.

The conclusions about the greater importance of maternal than paternal mental health seem somewhat over stated. Father’s mental health is important because it impacts mother mental health which then impacts child mental health. For instance, in the cross sectional analysis, mothers had highest bridge but the observed differences were non-significant

(except loss of confidence and child internalising). Please consider how strongly this point is made in the discussion.

The age finding is over stated because the age group analysis does not compare like with like. It could be entirely driven by changes in the methodology i.e. shared method variance. The statement on P16 therefore needs revising to reflect that. The authors do note this limitation but then re-state the age-difference again later in the discussion.

VERSION 1 - AUTHOR RESPONSE

Reviewer 1

Comments to the authors	Response
Major points	
P(age)8:l(ine)23 - The Authors claim that a dimensional reduction of the original data set is necessary "to avoid a model with too many variables". They aggregate several SDQ items together based on previous studies. GHQ-12 items are also aggregated.<br><br>There are not many details regarding SDQ scale but GHQ-12 is a "a 12-item instrument with a 4-point response scale". If I understood correctly, GHQ-12 scale should range between 0 and 48.<br><br>Could the Authors clarify why they have chosen a Poisson link function (P9:l11) for the marginal distribution of each category made of aggregated items in GHQ-12? The marginal distribution (as well as in SDQ) should be instead a Binomial distribution given the bounded range of the scale. For instance, "loss of confidence range" ranges between 0 and 8.<br><br>From a theoretical viewpoint, approximating Binomial(n, p) with Poisson is valid if n is large and np is small. However, taking "loss of confidence range" category as an example, n = 8 and the probability of success p should be extremely small for the validity of the approximation. Is this assumption on p correct in the real data application?<br><br>From a practical viewpoint, it would be interesting to visualise the marginal distribution of the categories and all pairs correlation (for instance by using the function ggpairs in GGally R package). Moreover, a QQ plot of the theoretical quantiles of a Poisson distribution	We thank the reviewer for this insightful comment. First of all, we expanded our description of both the GHQ-12 and SDQ measures on e.g. page 6, paragraph 4, and page 7, paragraph 1:<br>"The SDQ is a 25-item instrument with a 3-point response scale, measuring five domains: emotional symptoms, peer relationships, conduct problems, hyperactivity or inattention, and pro-social behaviours. Each domain was assessed using five items."<br><br>We acknowledge that the mention of a Poisson link function was an oversight carried over from earlier versions of the manuscript. In the current analysis, we assumed Gaussian distributions for all aggregated variables, based on four considerations. First, psychometric evidence suggests that variables derived from the sum of Likert-scale items approximate continuous distributions (Bishop & Herron, 2015; Carifio & Perla, 2008). Second, parametric tests are suggested to be sufficiently robust for analysing Likert-scale responses. Studies using simulated data have shown that parametric tests produce accurate estimates even when the normality assumption is violated to a considerable degree (Carifio & Perla, 2008; Norman, 2010). Third, when we did fit network models for the cross-sectional analysis using Poisson and binomial link functions, there were problems with model convergence. Lastly, the software for the longitudinal network models do not allow for non-Gaussian distributions.

versus the empirical quantiles should clarify the validity of the Poisson approximation.<br><br>In the longitudinal network analysis (P10:l12), what is the marginal distribution of the "family emotional symptoms" random variables?	In light of the reviewer's suggestion, we visualised the marginal distributions of all variables using the "ggpairs" function from the GGally R package. While the assumption of normality approximately held for some variables (e.g. children's externalising behaviours), we observed deviations from normality in others (e.g. parents' loss of confidence). Nevertheless, given that treating Likert-scale data as continuous is common in psychometric modelling, and that Gaussian methods have been shown to be relatively robust to violations of normality when the number of categories is small (Norman, 2010; Rhemtulla, Brosseau-Liard & Savalei, 2012), we retained the current analytic approach, but recognised this as a limitation.<br><br>We clarified the distributional assumptions and included visualisations of all variable distributions in the supplementary material, as noted in the main manuscript on page 8, paragraph 2:<br>"Derived variables based on the sum scores of the SDQ and GHQ-12 items were treated as continuous, in line with empirical evidence that aggregated Likert scales approximate continuous distributions (Bishop & Herron, 2015; Carifio & Perla, 2008). The marginal distributions of all variables were visualised and are provided in online supplementary appendix S2. Some approximated normality (e.g. children's externalising behaviours), whereas others deviated from it (e.g. parents' loss of confidence). Given parametric tests remain sufficiently robust when analysing Likert-scale data despite violations of the normality assumption (Carifio & Perla, 2008; Norman, 2010), we retained a Gaussian specification for all variables."<br><br>Similarly, in the longitudinal panel network analysis, all variables were treated as continuous. This was also partially due to software availability. Treatments of ordinal data in network approaches are still suboptimal (Borsboom et al., 2021). In fact, our ordinal/binary specification failed to converge, making the continuous approach the only practicable option. This approach is consistent with prior published studies that have applied

	panel network models to psychological symptom data using identical SDQ items (e.g. Panayiotou, Black, Carmichael-Murphy, Qualter, & Humphrey, 2023[1]). We have clarified this modelling assumption in the revised manuscript on page 9, paragraph 3: "Data were treated as continuous…"<br><br>Accordingly, we have added the following to the limitations in the discussion to reflect our consideration of the normality assumption on page 17, paragraph 2:<br>"Another limitation was that in the analysis, we treated the Likert items as approximately continuous and modelled them under a Gaussian assumption, partially due to software availability, as treatments of ordinal data in network approaches remain suboptimal (Borsboom et al., 2021). Treating Likert-scale data as continuous is common in psychometric modelling (Carifio & Perla, 2008; Sullivan & Artino, 2013), and Gaussian methods have been shown to be relatively robust to violations of normality when the number of categories is small (Norman, 2010; Rhemtulla, Brosseau-Liard, & Savalei, 2012). Nonetheless, this approach assumes equal spacing between response options and may bias parameter estimates."
P9:l7 - "Data from all available waves were used, with single observations randomly selected for families with repeated measures".<br><br>If I understood correctly, all data from all available waves were used in the cross-sectional analysis. However, per family, observations from all available waves are not independent (their temporal dependencies are modelled subsequently by using a vector autoregressive model), so graphical models which assume independence of the observations cannot be applied straightforwardly. The Authors recognised this problem when they sampled at random one observation from families with repeated measures.<br><br>Can the Authors clarify this point regarding the non-independence of the observations? Is there	In the cross-sectional analysis, we aimed to maximise sample size for statistical power while maintaining the assumption of independent observations. To address the issue of non-independence arising from repeated measures within families, we randomly selected a single observation per family across all available waves.<br><br>We agree that analysing only wave 1 would ensure strict temporal independence. However, our primary interest in the cross-sectional analysis was to estimate network structures across families, rather than modelling temporal change or focusing solely on baseline patterns. The current approach enabled us to retain more data while respecting the independence assumption.

[1] Panayiotou, M., Black, L., Carmichael-Murphy, P. *et al.* Time spent on social media among the least influential factors in adolescent mental health: preliminary results from a panel network analysis. *Nat. Mental Health* **1**, 316–326 (2023). https://doi.org/10.1038/s44220-023-00063-7

any way to account for the random effects produced by the longitudinal nature of the data and the presence of repeated measures?<br><br>Would it have been better to analyse the data only at wave 1 and use it as a baseline/reference graphical model?	We have clarified this rationale in the revised manuscript on page 7, paragraph 4:<br>“Data from all available waves were used, with single observations randomly selected for families with repeated measures. This approach allowed us to maximise sample size while maintaining temporal independence. ”
P9:l57 - The Authors stratify the data in the cross-sectional analysis by sex and age of the children, and family incomes. However, did the Authors correct for these covariates in the overall cross-sectional graphical model analysis? These covariates can produce spurious correlations and misleading results between the categories if not accounted for.<br><br>Are in the data set other covariates besides sex and age of the children, and family incomes that should be considered?<br><br>Moreover, when sex is used to stratify the data set, are the effects of age of the children and family incomes removed? Similarly when age of the children categories are used for the stratification.<br><br>In the longitudinal network analysis (P10:l12), family incomes covariate presumably changes over waves/time. How should it be treated to remove its effect?	We thank the reviewer for this insightful comment. In the current analysis, our primary aim was to provide an overall picture of the interconnections among mental health domains within and across family members, as well as to explore how these network structures vary across different subgroups (e.g. by child’s sex, age, and family income).<br><br>Rather than testing specific hypotheses, our approach was data-driven, aiming to characterise the structure of mental health networks in families. Future research could build on these findings to test specific hypotheses, such as whether links in the graph are moderated or mediated by certain variables (e.g. socio-economic status), under appropriate confounding control.<br><br>We acknowledge that not adjusting for covariates may introduce confounding and have clarified this limitation in the revised manuscript on page 17, paragraph 3:<br>“Moreover, this study adopted a data-driven approach and did not control for demographic or socio-economic covariates. While this aligned with our aim to characterise the overall structure of mental health networks in families, some of the associations could arise due to their shared relationship with confounders that future hypothesis-driven research should address. ”
Minor points	
P(age)4:l(ine)15: Please remove the term “causal”. Unless the Authors claim Granger causality, graphical models alone cannot establish causal dependency relations between time series.	Thanks, this has been removed
P9:l11: In the cross-sectional analysis, please provide more information regarding the mgm R package function used and its parametrisation (if different from the default values) as well as for the bootnet and networktools packages employed in the same analysis.	Addressed the mgm package on page 8, paragraph 2:<br>“…we retained a Gaussian specification for all variables. Networks were fitted using Mixed Graphical Models using the ‘mgm’ package in R. … All other parameters set to their default

	values.”<br><br>Addressed the bootnet package on page 8, paragraph 3:<br>“… estimated using the bootstrap procedure implemented in the R-package ‘bootnet’ (1,000 bootstraps; all other parameters set to default).”<br><br>Addressed the networktools package on page 9, paragraph 1:<br>“Bridge nodes’ centrality (bridge strength and bridge betweenness) was calculated using the R-package ‘networktools’, with default settings.”
P9:l5: It is unclear what the Authors mean by “independent correlations between nodes”. Do they mean a correlation graph?	Clarified and expanded on page 7, paragraph 4:<br>“Edges between nodes reflected partial correlations, indicating conditionally independent associations after controlling for all other nodes in the network.”
P9:l11: In the longitudinal analysis, please provide more information regarding which psychonetrics R package function has been used and its parametrization (if different from the default values).	Addressed and clarified on page 9, paragraph 3:<br>“…and models were estimated using the robust full-information maximum likelihood estimator using the R-package ‘psychonetrics’ and visualised with ‘qgraph’. … Parameters were set to their default values unless otherwise specified in the model development procedure below.”

Reviewer 2

Comments to the authors	Response
Introduction and discussion	
Quite a bit of the rationale for the study outlined in the introduction is based on the identification of intervention targets and a broad range of intervention studies are mentioned. Some of this rationale seems slightly over-stated because: many intervention targets for emotional problems are already known e.g. family therapy, CBT for depression in children. Moreover, some of the evidence on P4 refers to interventions for very young children in the section where it is highlighted that interventions that involve parents may be more effective. Relatively few studies are cited here and it’s also not clear to what extent this applies to the full age range examined in the study. Please consider if these statements are needed in the establishing the rationale for the	We appreciate this thoughtful comment. After consideration, we agree that some of the intervention-focused discussion in the introduction may have overstated the rationale. In response, we have removed the paragraph referencing early-years parent support interventions (on page 4), as this was less relevant to the age range and aims of our study.<br><br>We revised the text to more directly support the theoretical framing that the family system can serve a key context through which its members’ mental health difficulties may be shaped and addressed. In doing so, we incorporated more targeted evidence on the

study. These points seem more like discussion points.	effectiveness of family-based interventions on page 4, paragraph 2:<br>"There is supported by the effectiveness of family-based approaches (e.g. family therapy, parent training) for families of young people with common mental health problems (e.g. mood disorders, externalising behaviours) where children are the primary focus of concern (Carr, 2025)."
P4 – the authors say "with the family serving as a key instrument of change." This statement is not justified. Please either rephrase or justify with a citation.	We have clarified on this point on page 4, paragraph 2:<br>"As the immediate environment that shapes and maintains young people's mental health, it is reasonable to assume the family serves as a key instrument of change for this."
P4 – an incomplete argument "However, to optimize……". Please state which aspects of mental health and family relationship are most important for what.	Clarified and expanded on page 4, paragraph 2:<br>"However, to optimise interventions, whether at the public health level or for individuals, a better understanding is needed of which family relationships and dimensions of mental health are most important for effecting positive change in the mental health of both children and their parents."
In the introduction there is a relatively long section discussing the role of genetic influences in intergenerational transmission (P5). This seems misplaced because this study cannot control for genetic influences. Also, the range of potential genetic influences at play is not considered such as shared genes but also processes such as passive gene environment correlation. Finally inter-generational processes differ for externalising and internalizing problems. This latter point suggests that the intervention targets are likely to be different for different aspects of mental health. Please consider whether this section is needed in the introduction. This seems more like a discussion point.	Agreed. The discussion of genetic influences has been removed from the introduction
The authors may wish to mention family systems theory in introduction	Our analysis was indeed grounded in family systems theory, which was introduced at the beginning of the manuscript (i.e. the first two sentences of the introduction). We have now made this point more explicit in the revised manuscript on page 4, paragraph 1:<br>"Family is a dynamic, interconnected, and multifaceted system in which members are interdependent and unavoidably embedded. This family system theory implies the interdependence of members in the family system, meaning that the mental health and

	well-being of one member can have a profound effect on another (for better or worse; Cox & Paley, 1997)."
P5 the authors say ….“this makes it difficult to disentangle the specific…..” That is true but it’s not clear how the present study addresses that. It’s therefore not clear if this statement is needed.	Agreed. This discussion was related to genetic influences, which has been removed from the introduction
The rationale for focusing on emotional problems seems a little odd because it’s a rationale for focusing on emotional problems completely. Whereas one analysis focuses on emotional problems (longitudinal) and one does not. Perhaps re-think the justification?	Clarified and expanded on page 5, paragraph 3:<br>“By using mental health measures from children and parents, we first aim to describe unique associations between broad domains of family members’ mental health using cross-sectional networks. This was intended to provide an overall picture of the family mental health network structure. Young people have experienced a sharp increase in emotional symptoms, with a relative rise of 63% from 2004 to 2017 (Taxiarchi et al., 2025), while trends in other symptoms (e.g. conduct behaviours) have been less pronounced or remained relatively stable (Pierce et al., 2025). Therefore, we then focus on specific symptoms of emotional problems using a longitudinal network to model a directional network between family members. This allowed us to model the dynamic pathways through which emotional symptoms may emerge and evolve in the family context.”
Missing details	
Are multiple children per family included? If yes, how is the non-independence accounted for.	We thank the reviewer for raising this point. To clarify, multiple children from the same family were included in the analysis if they met the selection criteria. For each child, one observation was randomly selected across all available waves to ensure temporal independence. In our sample, the majority (80%) of children came from different families. To account for potential non-independence due to the inclusion of siblings, we conducted a sensitivity analysis by randomly selecting only one child per family. The results were largely consistent with our main findings (see revised supplementary appendix S1), supporting the validity of our approach. This point has been clarified in the revised manuscript on page 7, paragraph 4, and page 8, paragraph 1:<br>“We included all children and young people

	who met the selection criteria, including multiple children from the same family, by randomly selecting one observation for each child. 79.8% of children in the sample came from different families. We conducted a sensitivity analysis by randomly selecting only one child per family. The results remained largely consistent with the main findings (see online supplementary appendix S1), supporting the validity of the current approach."
Please describe the GHQ in more detail – what is it thought to measure P7.	We have clarified on page 7, paragraph 2: "…the GHQ-12 measures general mental distress and, according to Shevlin & Adamson (2005), can be divided into three subscales to reflect: emotional symptoms (4-items), social dysfunction (6-items), and loss of confidence (2-items)."
There is no explanation or justification of the subgroup analyses on P8. Please add to P8.	Clarified on page 9, paragraph 2: "Finally, it is reasonable to assume that the structure of family mental health networks may vary across different subgroups, such as by the gender and age of children and young people, as well as household income (Hughes & Gullone, 2008; Leinonen, Solantaus, & Punamäki, 2003). Subgroup networks were then analysed for…"
How is missing data handled in the longitudinal analysis.	The analyses were based on complete cases, and this point has been added in the revised manuscript on page 9, paragraph 3: "The analyses were based on complete cases."
Queries	
The authors interpret the finding from the symptom of being overwhelmed as being driven by anxiety. I am not sure that feeling overwhelmed is an anxiety symptom as per discussion. It could be a symptom of anxiety, or it could driven by hopelessness or functional impairment. I would suggest broadening the focus in the discussion of this result i.e. it's hard to justify that it's only anxiety.	We agree that feeling overwhelmed may not be uniquely indicative of anxiety and could reflect a broader profile of emotional distress. However, we would like to clarify that in our longitudinal analysis, feeling overwhelmed showed strong links to later emotional symptoms (e.g. unhappiness), rather than being strongly driven by them. This suggests that it may function more as a symptom than a downstream consequence in this context. We have updated the discussion to reflect this more nuanced interpretation on page 14, paragraph 3 and page15, paragraph 1: "Mothers' experiences of feeling overwhelmed and under strain led children to experience worry in subsequent waves, which, in turn, activated children's symptoms of anxiety (e.g. nervousness and fear) and

	depression (e.g. unhappiness). Although feelings of being overwhelmed and strained could be seen as symptoms of anxiety (American Psychiatric Association, 2022) or as consequences of other disorders (e.g. depression or functional impairment), the direction of the associations from our panel network analysis suggests these feelings function primarily as originating symptoms rather than arising from other symptoms measured in the GHQ-12. For example, maternal feelings of overwhelm strongly predicted later depressive symptoms (e.g. unhappiness), with a much stronger estimated association in this direction than in the reverse. Therefore, while much research has focused on maternal depression, our findings highlight the critical role of maternal anxiety and stress in shaping both maternal and child mental health. ”
P14. The authors say that “women tend to experience more enduring mental health problems than fathers”. I’m not sure this is justified. Please justify or rephrase.	Agreed. This statement was not relevant to the current analysis and has been removed from the revised manuscript.
On P14 the authors say that the association between fathers and children may be weaker because it includes step-children. Are the authors able to provide some detail on this e.g. are they able to report the likely % of step-father families or test this?	Yes we were able to examine this. In our sample, 89% of children in blended families lived with their biological mother. This detail has been added to the revised manuscript on page 14, paragraph 2:<br>“Indeed, in our sample, 88.5% of children in blended families lived with their biological mother.”
Discussion and limitations	
An important limitation not mentioned is that only 75% of the sample with children in Understanding Society met the inclusion criteria. It seems likely that there is socio-economic patterning of complete trio data (e.g. other studies have shown ascertainment bias for complete father, mother and child trios when collecting DNA samples e.g. Westhttps://pubmed.ncbi.nlm.nih.gov/12399949/ [pubmed.ncbi.nlm.nih.gov]). Please mention as a limitation.	Thanks, this limitation has been added to the revised manuscript on page 16, paragraph 4, and page 17, paragraph 1:<br>“Second, we included family structures where a triad of children and two parents was present, which accounted for 75.6% of households with children in UKHLS. It is likely that the availability of complete triadic data is socioeconomically patterned. Previous research has demonstrated phenotypic differences between children from complete trios versus duos (i.e. missing one parent), with trios showing lower levels of psychopathology and conduct disorder symptoms (West et al., 2002). This selective inclusion could result in a sample that is not fully representative of the broader population.”

The longitudinal data set sample size is less than half that of the cross-sectional sample size. This should be mentioned as a limitation. As does attrition and selective attrition.	Thanks, this limitation has also been added to the revised manuscript on page 17, paragraph 1:<br>"The longitudinal network analysis included a smaller number of families, due to sample attrition and the exclusion of older children who aged out of the youth sample across waves. This reduced sample size may have limited the statistical power to detect associations and introduced additional bias if attrition was non-random."
The conclusions about the greater importance of maternal than paternal mental health seem somewhat overstated. Father's mental health is important because it impacts mother mental health which then impacts child mental health. For instance, in the cross sectional analysis, mothers had highest bridge but the observed differences were non-significant (except loss of confidence and child internalising). Please consider how strongly this point is made in the discussion.	We fully acknowledge the importance of paternal mental health and do not intend to undermine its role. As the reviewer mentioned, our cross-sectional analysis revealed close associations between maternal and paternal mental health domains. We acknowledge that this has two potential implications: (1) paternal mental health may influence child mental health indirectly via its effect on maternal mental health, and (2) fathers may serve as a valuable point of intervention to improve family mental health. These points have been mentioned in the discussion of the cross-sectional network results on page 14, paragraph 2, and the clinical implications on page 17, paragraph 3.<br><br>However, in the longitudinal analysis, we did not observe significant associations between fathers' emotional symptoms and either mothers' or children's emotional symptoms when all family members were considered together. Therefore, we feel it is important to retain one paragraph interpreting the finding that the link between maternal and child symptoms appears to be more robust in this context.<br><br>In light of the reviewer's comment, the tone has been revised to better reflect our consideration of the father's role on page 13, paragraph 3:<br>"Indeed, in the cross-sectional network, we observed associations between each of the mothers' and fathers' mental health domains, underscoring the relevance of paternal mental health within the family mental health system."
The age finding is over stated because the age group analysis does not compare like with like. It	Agreed. The discussion of clinical implications based on age subgroup analyses originally on

could be entirely driven by changes in the methodology i.e. shared method variance. The statement on P16 therefore needs revising to reflect that. The authors do note this limitation but then re-state the age-difference again later in the discussion.	page 16 has been removed.

Note: Besides the revisions based on reviewers' comments, we also updated the reference format in the manuscript to better align with the BMJ Open guidelines.

VERSION 2 - REVIEW

Reviewer **1**

Name **Bottolo, Leonardo**

Affiliation **MRC Biostats**

Date **23-Aug-2025**

COI

The Authors replied carefully to my comments.

Could you please add this reference

Han Liu, John Lafferty and Larry Wasserman. The Nonparanormal: Semiparametric estimation of high-dimensional undirected graphs, Journal of Machine Learning Research, 10, 2295—2328, (2009).

regarding graphical models where the assumption of normality does not hold. Can they also add a sentence in the Discussion regarding the need for readily available software that can perform such analysis?

VERSION 2 - AUTHOR RESPONSE

Reviewer 1

Comments to the authors	Response
The Authors replied carefully to my comments.<br><br>Could you please add this reference<br><br>Han Liu, John Lafferty and Larry Wasserman. The Nonparanormal: Semiparametric estimation of high-dimensional undirected graphs, Journal of Machine Learning Research, 10, 2295—2328, (2009).	We appreciate the reviewer's helpful recommendation. We have now cited the suggested reference when discussing the limitation of applying a Gaussian assumption in contexts where normality does not hold, and we have also mentioned the need for readily available software that can perform such analyses on page 17, paragraph 2: "Alternative semiparametric approaches, such

regarding graphical models where the assumption of normality does not hold. Can they also add a sentence in the Discussion regarding the need for readily available software that can perform such analysis?	as nonparametric extensions of undirected graphical models,48 have been proposed to relax the normality assumption when estimating undirected graphs. However, their wider uptake in applied research is likely to depend on the availability of more accessible, user-friendly software." Reference 48 corresponds to Liu et al. (2009).

Note: Besides the revisions based on reviewers' comments, we also updated the manuscript to better align with the BMJ Open guidelines.
